# Micromotion Derived Fluid Shear Stress Mediates Peri‐Electrode Gliosis through Mechanosensitive Ion Channels

**DOI:** 10.1002/advs.202301352

**Published:** 2023-07-30

**Authors:** Alexandre Trotier, Enrico Bagnoli, Tomasz Walski, Judith Evers, Eugenia Pugliese, Madeleine Lowery, Michelle Kilcoyne, Una Fitzgerald, Manus Biggs

**Affiliations:** ^1^ SFI Research Centre for Medical Devices (CÚRAM) University of Galway Galway H91 W2TY Ireland; ^2^ Galway Neuroscience Centre University of Galway Galway H91 W2TY Ireland; ^3^ Department of Biomedical Engineering Faculty of Fundamental Problems of Technology Wrocław University of Science and Technology Wroclaw 50‐370 Poland; ^4^ School of Electrical and Electronic Engineering University College Dublin Dublin 4 Ireland; ^5^ Carbohydrate Signalling Group Discipline of Microbiology University of Galway Galway H91 W2TY Ireland

**Keywords:** astrogliosis, glial scar, ion channels, mechanosensing, neuroelectrodes

## Abstract

The development of bioelectronic neural implant technologies has advanced significantly over the past 5 years, particularly in brain–machine interfaces and electronic medicine. However, neuroelectrode‐based therapies require invasive neurosurgery and can subject neural tissues to micromotion‐induced mechanical shear, leading to chronic inflammation, the formation of a peri‐electrode void and the deposition of reactive glial scar tissue. These structures act as physical barriers, hindering electrical signal propagation and reducing neural implant functionality. Although well documented, the mechanisms behind the initiation and progression of these processes are poorly understood. Herein, in silico analysis of micromotion‐induced peri‐electrode void progression and gliosis is described. Subsequently, ventral mesencephalic cells exposed to milliscale fluid shear stress in vitro exhibited increased expression of gliosis‐associated proteins and overexpression of mechanosensitive ion channels PIEZO1 (piezo‐type mechanosensitive ion channel component 1) and TRPA1 (transient receptor potential ankyrin 1), effects further confirmed in vivo in a rat model of peri‐electrode gliosis. Furthermore, in vitro analysis indicates that chemical inhibition/activation of PIEZO1 affects fluid shear stress mediated astrocyte reactivity in a mitochondrial‐dependent manner. Together, the results suggest that mechanosensitive ion channels play a major role in the development of a peri‐electrode void and micromotion‐induced glial scarring at the peri‐electrode region.

## Graphical abstract

This study reveals that peri‐electrode void progression is mediated by fluid flow shear stress, which can be replicated using parallel‐plate flow chambers to induce neuroelectrode glial scarring in vitro. Astrocyte PIEZO1 (piezo‐type mechanosensitive ion channel component 1) and TRPA1 (transient receptor potential ankyrin 1) are upregulated at the peri‐electrode region in response to electrode micromotion. PIEZO1 pharmaceutical activation diminishes shear stress‐induced gliosis while its inhibition exacerbates gliosis and reduces mitochondrial functions.

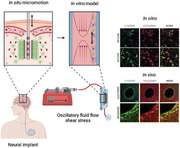

## 1 Introduction

Technical innovations in bioelectronic technologies have facilitated the development of smaller, smarter, and less invasive diagnostic and therapeutic bioelectronic devices, with current research focused on increasing the long‐term performance of electrode‐based technologies in vivo. The development of electrically stable, tissue‐interfacing technologies represents a paradigm shift in the treatment of chronic disease and real‐time health‐monitoring and an understanding of the mechanical instigators and contributors to the proinflammatory events which occur at the peri‐electrode region is critical to mitigate the detrimental effects of implant encapsulation on neural recording quality and stimulation stability.^[^ ^1^, ^2^ ^]^

Chronic neuroelectrode function is often compromised by the foreign body response, resulting in peri‐electrode fibrosis and encapsulation, a process termed reactive gliosis, which can result in the loss of neuroprosthestic functionality.^[^ ^1^ ^]^ The associated inflammatory processes of gliosis are thought to be mediated principally by resident astrocytes, microglia and NG2 glia cells,^[^ ^3^ ^]^ which adopt a reactive and proliferative phenotype in response to localized tissue damage, leading to neuronal cell loss and the deposition of a collagen and chondroitin sulfate rich fibrous tissue.^[^ ^4^, ^5^ ^]^

Initially, peri‐electrode inflammation is triggered by mechanical trauma to blood vessels, capillaries, and cells during implant insertion, originally proposed as the principal initiator of glial scar development.^[^ ^6^, ^7^ ^]^ Attempts to attenuate implantation‐associated trauma have focused on modifying the electrode chemistry,^[^ ^8^, ^9^ ^]^ geometry^[^ ^10^, ^11^ ^]^ or by coating the neural implant with anti‐inflammatory molecules to improve device integration.^[^ ^12^, ^13^, ^14^ ^]^ However, studies have shown that this initial inflammatory response to the mechanical damage of implantation is quickly resolved upon device removal.^[^ ^6^, ^15^ ^]^ Furthermore, a number of studies have identified a significant role of the mechanical properties mismatch between stiff neural probes and the relatively soft brain tissue in promoting reactive gliosis,^[^ ^16^, ^17^ ^]^ leading to the development of softer or mechanically adaptive neural electrodes.^[^ ^18^, ^19^, ^20^ ^]^

A growing body of evidence has further showed that relative micromotion at the electrode/tissue interface arising from respiration, pulsatile blood flow, and physical movement,^[^ ^21^, ^22^, ^23^ ^]^ exposes the peri‐electrode tissues to cyclic shear stresses,^[^ ^27^, ^28^, ^29^ ^]^ which exacerbates the inflammatory state initially primed by neuroelectrode insertion,^[^ ^30^, ^31^ ^]^ and promotes glial scarring in vivo.^[^ ^24^, ^25^, ^26^ ^]^ Critically, under acute conditions the electrode interface is biologically and electrically unstable and the peri‐electrode region is characterized by the development of a progressive fluid filled space, which appears to reach a steady state at ≈3× the electrode diameter.^[^ ^24^, ^25^ ^]^

Despite insightful advances in mechanobiology, the mechanism by which cells of the central nervous system (CNS) can sense mechanical variations and the mechanotransductive molecular pathways which initiate gliosis are not fully understood. Recently, mechanosensitive (MS) ion channels have come to the forefront of neuromechanobiology and have been shown to undergo activation or differential expression in response to mechanical stimuli within the CNS,^[^ ^32^ ^]^ with important functions in blood‐brain barrier maintenance,^[^ ^33^ ^]^ neural differentiation,^[^ ^34^, ^35^ ^]^ nociception,^[^ ^36^, ^37^, ^38^ ^]^ neural–glia communication,^[^ ^39^ ^]^ and glia activation.^[^ ^40^, ^41^, ^42^, ^43^, ^44^, ^45^ ^]^ Critically, MS channel expression is perturbed in neurodegenerative disorders^[^ ^46^ ^]^ including Alzheimer's disease,^[^ ^45^, ^47^ ^]^ myelination disorders,^[^ ^48^, ^49^, ^50^ ^]^ migraine,^[^ ^51^, ^52^, ^53^ ^]^ and glioma.^[^ ^54^, ^55^, ^56^ ^]^

In order to gain insight into the role of cyclic mechanical forces in the onset and evolution of peri‐electrode gliosis, recent studies have described the development of cell culture models, which mimic physiologically relevant neuroelectrode micromotions and which have demonstrated partial success in reproducing the processes of gliosis in vitro.^[^ ^57^, ^58^, ^59^ ^]^ It follows that the development of pathophysiological models of the peri‐implant micromechanical environment that replicate the glial scarring process may provide valuable tools for further understanding the mechanobiology of neural populations and promote the development of novel approaches to mitigate the foreign body response to neural implants.

In this work, we describe a novel computational model of the peri‐electrode region, encompassing solid, liquid, and viscoelastic elements, representing the neuroelectrode, the fluid filled peri‐electrode space and the surrounding neural tissues, respectively. We proceed to show that in a chronic setting, micromotion‐induced peri‐electrode fluid shear stresses occur in the mPa range and that rat ventral mesencephalic E14 embryonic cells exposed to this level of fluid shear through a parallel‐flow chamber undergo gliosis‐associated cellular processes in vitro. It was further observed that mPa fluid shear stress induced the overexpression of two mechanosensitive ion channel receptors in vitro, piezo‐type mechanosensitive ion channel component 1 (PIEZO1) and transient receptor potential ankyrin 1 (TRPA1), which was replicated in an in vivo neural probe encapsulation scar model. Moreover, we show that the inhibition of PIEZO1 triggers and increases gliosis response, and hinders mitochondrial functions in vitro, in ventral mesencephalic cells. Conversely, chemically induced activation of the PIEZO1 receptor stalled the neuroinflammatory response of the culture when subjected to shear stress conditions. Together these results implicate mPa shear stress as a potent mediator of neural mechanotransduction and offer new insight into the mechanism of neuroelectrode‐induced foreign body reaction.

## 2 Results

### 2.1 Neuroelectrode Micromotion Induces Oscillatory Fluid Flow and Imparts Millipascal Shear Stress to Peri‐Electrode Tissues

**Figure 1:**
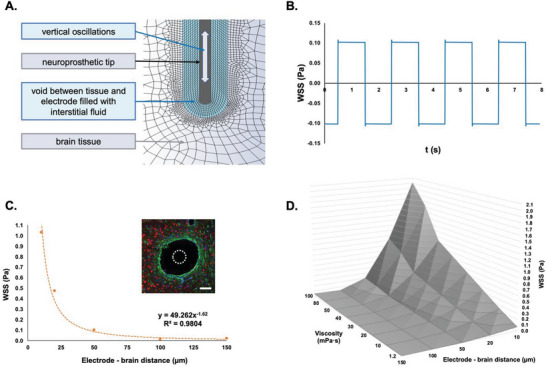
Neuroelectrode micromotion generates oscillatory fluid flow shear stress as determined by the peri‐electrode void volume and fluid viscosity. A) Schematic of the model configuration for FVM analysis, including neural probe and mesh. B) The simulated electrode movement induced oscillatory WSS at the brain tissue surface with constant frequency and amplitude. C,D) Representative histological section of electrode implantation in the STN of adult rats stained for GFAP (green), NeuN (red), and DAPI (blue) (scale bar = 100 µm), the dotted white circle symbolises the electrode position and diameter (100 µm) showing the peri‐electrode space created around the probe. The WSS magnitude decreased as the distance between the electrode and the brain tissue increases due to peri‐electrode space expansion and with decreasing viscosity of the fluid within the peri‐electrode space.

Current numerical models of brain micromotion to understand and predict local tissue damage arising from implanted neuroprosthetics assume complete electrode tissue apposition for the analysis of tissue strain.^[^ ^18^, ^60^, ^61^, ^62^, ^63^, ^64^ ^]^ However, in vivo, neuronal death and progressive regression of nervous tissues from the electrode periphery suggest that the electrode/tissue interface is characterized by a dynamic and expanding peri‐electrode fluid‐filled void, that reaches equilibrium when it obtains a diameter of ≈2–4 times that of the implanted neuroelectrode, irrespective of electrical stimulation parameters.^[^ ^18^, ^19^, ^24^, ^25^, ^65^ ^]^ Therefore, a more representative finite volume model (FVM) of the electrode/brain tissue interface was developed to assess the magnitudes of fluid shear stress on the peri‐electrode tissues, imparted through electrode micromotion within the void (**Figure** **1**).

In this model, the electrode oscillated within a fixed diameter fluid‐filled cavity, with constant frequency and amplitude resulting from respiratory activity (amplitude of 30 µm peak‐to‐peak with a frequency of 0.5 Hz arising from respiration) (Figure 1A,B). Electrode micromotion was noted to create a flow of the interstitial fluid, which generated wall shear stress (WSS) at the tissue interface (Figure 1B) as a function of the electrode–brain tissue distance (Figure 1C), and the interstitial fluid viscosity (Figure 1D).

The average WSS magnitude was observed to vary from 0.0006 Pa for an electrode–brain distance of 150 µm and a viscosity value of 1.2 mPa s, to a shear stress of 2.0167 Pa for an electrode–brain distance of 10 µm and a viscosity of 100 mPa s (Figure 1D). Additionally, when considering the interstitial fluid viscosities reported in the literature for healthy brain tissues (1–50 mPa s),^[^ ^66^ ^]^ it was observed that the WSS imparted by fluid flow shear within the peri‐electrode fluid space increased considerably as the electrode–tissue distance was reduced to less than 10 µm (Figure 1C,D).

Specifically, assuming a 200 µm peri‐electrode cavity (twice the electrode diameter), an electrode–brain tissue distance equal to 50 µm (on both electrode sides) and an interstitial fluid viscosity of 50 mPa s, the resulting WSS arising from electrode micromotion was ≈0.1 Pa (Figure 1C,D). As the volume of the peri‐electrode fluid space is observed to increase in vivo through dynamic radial expansion it can be inferred that a WSS > 0.1 Pa may result in astrocyte reactivity and neuronal loss, promoting expansion of the peri‐electrode fluid space (Figure 1C). With this hypothesis, a WSS of 0.1 Pa was chosen for the subsequent in vitro parallel plate flow chamber (PPFC) studies into astrocyte activation (Figures S1 and S2, Supporting Information).

Initially the capacity of the PPFC device to induce consistent millipascal fluid shear stresses on a cellular monolayer culture was assessed through computational fluid dynamic (CFD) analysis (Figure S1, Supporting Information), confirming theoretical assumptions that in a parallel plate laminar flow system fluid flow WSS depends on the volumetric flow rate *Q*, the dynamic viscosity *μ*, and channel dimensions (width *b* and height *h*) as described by Equation (1).^[^ ^67^ ^]^

(1) $$\tau_{w}=\frac{6Q\mu }{bh^{2}}$$

In this study, three volumetric flow rates corresponding to WSS of 0.1, 1, and 10 Pa were assessed in silico. As the *b*/*h* ratio of the microchannel device was equal to 120, in steady conditions, 0.5 Hz oscillatory flows produced highly homogeneous WSS (Figure S1C,D, Supporting Information) for all investigated flow regimes (Table S1, Supporting Information). Importantly, a flow rate of 4.49 mL.min^−1^ provided uniform shear conditions during the flow period (Figure S1G, Supporting Information) and resulted in a WSS value of 0.1 Pa (Figure S1F, Supporting Information), while the Reynolds number did not exceed 2.4.

Primary neural cells were extracted from the ventral mesencephalon of E14 rat embryos and seeded onto glass slides five days prior to exposure to millipascal shear stress. At day 0, the glass slide was placed in the parallel flow apparatus and exposed to an oscillatory fluid flow at 0.1, 0.5, and 1 Pa at 0.5 Hz for either 4 or 6 h in culture medium. Following shear stimulation, glass slides were subdivided into three equal pieces, and kept in culture until experiment endpoint for either PFA fixation or RNA/protein extraction (Figure S2, Supporting Information).

### 2.2 Oscillatory Fluid Flow Shear Stress Upregulates Markers of Astrogliosis and Reduces Neuronal Viability

**Figure 2:**
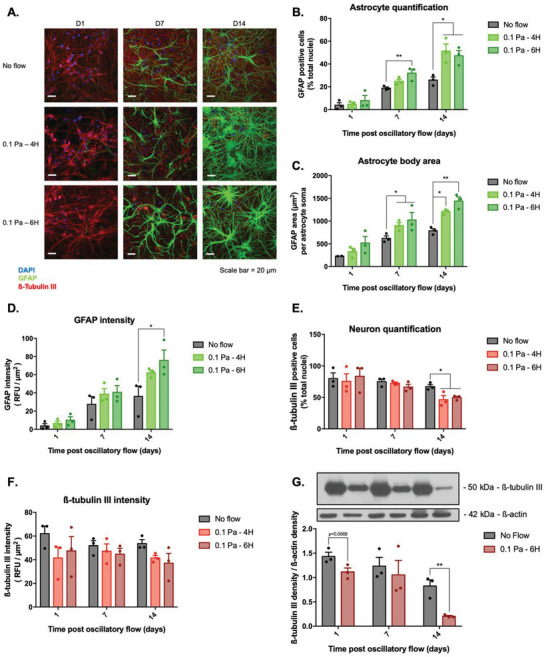
Oscillatory fluid flow shear stress promotes astrocyte reactivity and neuron death in ventral mesencephalic (VM) cells. A) VM cells were exposed to 0.1 Pa of fluid shear stress for 4 or 6 h and immunofluorescently stained for GFAP and ß‐tubulin III after 1, 7, and 14 days (scale bar = 20 µm; *n* = 3). B) Exposure to fluid shear stress increased astrocyte proliferation by approximately twofold at 7 and 14 days postflow stimulation. C) The average cell area of the astrocyte was also increased by ≈30–50% at both 7 and 14 days following oscillatory fluid flow stimulation. D) Oscillatory shear stress upregulated GFAP intensity by 40–50% in astrocytes 14 days poststimulation. After 14 days postexposure to oscillatory 0.1 Pa shear stress for 4 or 6 h, E) a significant decrease in the total number of neurons, and F) a reduction in ß‐tubulin III fluorescent intensity were observed. G) This reduction in ß‐tubulin III expression was confirmed by western blot analysis and a significant 65% decrease was observed in VM cells exposed to 0.1 Pa of fluid shear stress for 6 h by day 14. Data are represented as mean ± SEM (*n* = 3–4). One‐way ANOVA with Tukey post hoc test was performed. *, ** represent a statistically significant difference (*p* < 0.05) and (*p* < 0.01), respectively.

Increased astrocyte proliferation and GFAP expression, in association with increased nuclear and cell area are well‐established markers of astrogliosis in vitro.^[^ ^1^, ^6^ ^]^ To examine if the millipascal scale fluid shear stresses which arise from neuroelectrode micromotion can trigger astrocyte reactivity and neuron degeneration, we initially explored modulation of cell morphology and cytoskeletal biochemistry in astrocyte and neuronal populations in response to oscillatory shear stress in vitro (**Figure** **2**). Ventral mesencephalic (VM) populations were fluorescently stained for GFAP, ß‐tubulin III, and DAPI (astrocytes, neurons, and nuclei, respectively). Initial image analysis revealed that neuron and astrocyte populations exposed to shear stresses of 0.5 and 1 Pa at 0.5 Hz were nonviable after 24 h and these shear magnitudes were excluded from further study (Figure S3, Supporting Information). Conversely, VM cell populations exposed to fluid flow stimulation at 0.1 Pa at 0.5 Hz displayed a significant 70% increase in the astrocyte:neuron ratio (control 19% vs treated 32%; *p* = 0.009), after 7 days in culture following 6 h under flow conditions (Figure 2A).

This effect was further observed at 14 days postshear stress stimulation, where the astrocyte:neuron ratio was significantly increased by 98% (control 26% vs treated 51%; *p* = 0.0242) and 82% (control 26% vs treated 47%; *p* = 0.0482) under 4 and 6 h of fluid flow, respectively (Figure 2B). Shear stress also increased the astrocyte soma area, at day 7 postflow, by 45% (control 622 µm^2^ vs treated 901 µm^2^; *p* = 0.0483) under the 4 h flow conditions and by 65% (control 622 µm^2^ vs treated 1025 µm^2^; *p* = 0.0215) under the 6 h flow conditions.

At 14 days postflow stimulation, the astrocyte cell body area was also significantly increased by 54% (control 787 µm^2^ vs treated 1208 µm^2^; *p* = 0.0241) and by 83% (control 787 µm^2^ vs treated 1441 µm^2^; *p* = 0.0053) following 4 and 6 h of fluid flow shear stress, respectively (Figure 2C). Astrocytes also demonstrated a nuclear area increase of 34% (*p* = 0.0102) and 28% (*p* = 0.0314), following 14 days of oscillatory fluid flow stimulation for 4 and 6 h, respectively (Figure S4A, Supporting Information).

Finally, following exposure to 6 h of 0.1 Pa shear stress, a significant >100% increase (control 36 RFU µm^−2^ vs treated 76 RFU µm^−2^; *p* = 0.0203) in the fluorescent intensity of GFAP was observed after 14 days (Figure 2D), which was further confirmed by western blot analysis (Figure S4B,C, Supporting Information).

Conversely, 14 days following exposure to 0.1 Pa shear stress, the number of neurons significantly decreased by 30% (control 67% vs treated 46%; p = 0.0154) after 4 h of stimulation and by 26% (control 67% vs treated 50%; p = 0.0303) after 6 h of stimulation, relative to cells cultured under static control conditions (Figure 2E; Figure S4D, Supporting Information). In addition, a consistent negative trend in the overall ß‐tubulin III fluorescent intensity was observed in neuron populations exposed to all flow conditions and at all experimental timepoints, relative to neuron populations cultured under static control conditions (Figure 2F). To confirm the effect of the fluid flow shear stress on ß‐tubulin III expression in VM populations, western blot analysis was carried out on cell lysates, where a reduction in ß‐tubulin III protein expression was observed in all VM populations under flow conditions. Specifically following 6 h of flow, a significant reduction of 76% in ß‐tubulin III protein expression (control 0.83 vs shear‐exposed 0.20; *p* = 0.0082) was observed at 14 days postflow (Figure 2G; Figure S4E, Supporting Information).

### 2.3 Oscillatory Millipascal Fluid Flow Shear Stress Upregulates the Expression of Neuroinflammation and Brain Injury Markers

**Figure 3:**
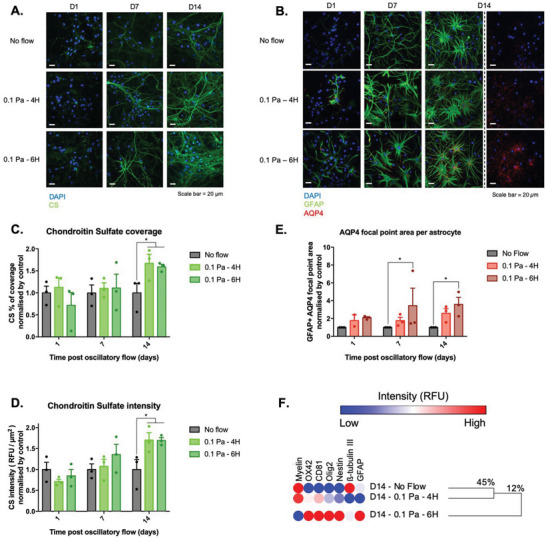
Exposing VM cells to oscillatory 0.1 Pa fluid shear stress upregulates indicators of astrogliosis, glial scarring, and brain injury in vitro. A,C,D) The fluorescence intensity and area of chondroitin sulfate (CS) staining, as well as B,E) the expression of water channel Aquaporin‐4 (AQP4), was significantly increased in cells exposed to flow shear stress conditions (scale bar = 20 µm; *n* = 3). F) A custom antibody protein microarray was used to derive a hierarchically clustered heatmap depicting binding intensities of whole cell lysate (*n* = 4). This analysis clustered together the static control condition with the 4 h stimulation condition, while setting out a single group with the 6 h shear stress condition, sharing only 12% similarity with the static and 4 h stimulation experimental conditions. Data are represented as mean ± SEM (*n* = 3). One‐way ANOVA with Tukey post hoc test was performed. * Represents a statistically significant difference of *p* < 0.05.

In the CNS, traumatic and neurodegenerative events are associated with upregulations in secondary neuroinflammatory markers including glycosaminoglycan chondroitin sulfate (CS)^[^ ^5^ ^]^ and the water channel aquaporin‐4 (AQP4).^[^ ^68^ ^]^ To determine if shear stress could similarly induce these upregulations in vitro, flow stimulated cultures were immunofluorescently stained for CS and AQP4 at 1, 7, and 14 days (**Figure** **3**A,B). Moreover, the proteomic expression of stimulated VM cells was further investigated using a custom protein array which targeted eight established markers of brain injury (Figure 3F).

It was observed that the percentage of VM cells expressing CS was increased by oscillatory fluid flow, at 14 days poststimulation by 67% (*p* = 0.0195) following 4 h of exposure to fluid shear stress and by 59% (*p* = 0.0333) following 6 h of exposure to fluid shear stress (Figure 3C). Upregulation in the expression of CS was also confirmed through quantification of the staining fluorescent intensity which was significantly increased by 70% in both 4 and 6 h treated cultures (*p* = 0.0276 and *p* = 0.0295, accordingly), 14 days after mechanical stimulation relative to control conditions (Figure 3D).

In addition, the VM astrocyte population exhibited upregulations in AQP4 expression (Figure 3B). In particular, cells subjected to 6 h flow condition displayed a significant ≈2.5‐fold increase at both day 7 (control 1 vs treated 3.44; *p* = 0.0374) and 14 days (control 1 vs treated 3.58; *p* = 0.0292) post oscillatory flow relative to VM cells cultured under static control conditions (Figure 3E).

At the protein level, hierarchical analysis of the binding intensity values obtained from a custom gliosis antibody array indicated the presence of two distinct protein expression clusters 14 days postflow (Figure 3F). The first, including the no‐flow control and the 4 h treatment condition, displayed 45% of similarity. While, the second cluster, including only the 6 h flow condition, presented only 12% of similarity with the no‐flow control and the 4 h treatment conditions.

The microarray analysis confirmed an increase in GFAP protein synthesis in VM cells subjected to 6 h flow conditions, and a decrease in the synthesis of ß‐tubulin III in cell populations subjected to 4 and 6 h of fluid flow shear stress. Furthermore, astrocyte reactivity was associated with a significant upregulation in the synthesis of Nestin, while downregulation of Myelin protein further indicated neuronal loss. Finally, the protein microarray revealed the overexpression of OX42 and CD81, proteins that become upregulated in activated microglia, as well as an increase in the expression of Olig2, a protein shown to have a neuroprotective role following brain injury. Together, these findings provide further evidence that the application of oscillatory fluid flow shear stress on VM cells is able to recapitulate the cellular processes of reactive gliosis in vitro.

### 2.4 Oscillatory Millipascal Fluid Shear Stress Promotes a Proinflammatory Phenotype, Neurodegeneration, and Modulates Mechanotransduction Processes in Neural Populations

**Figure 4:**
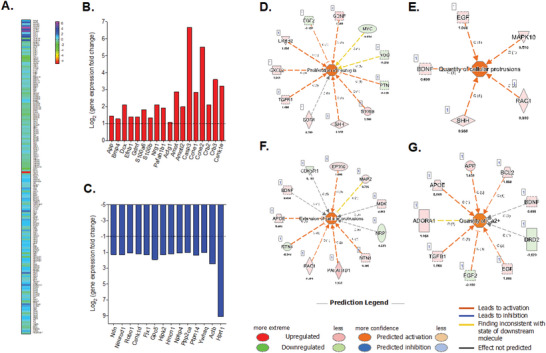
Exposing VM cells to oscillatory 0.1 Pa fluid shear stress induces glial cell proliferation and growth, neurodegeneration, and modulates neural function pathways. A) The genomic expression of VM cells following 6 h of flow shear stress in vitro was assessed using a qPCR array targeting 168 genes related to neurogenesis and the Hippo pathway. B) The 18 most upregulated and C) 14 most downregulated genes related to astrocyte reactivity, neurodegeneration, and brain injury are displayed in red and blue, respectively (Log_2_ fold regulation; dotted line representing the cut‐off of ≤2‐fold or ≥−2‐fold regulation, respectively; *n* = 4 pooled biological replicates). Qiagen Ingenuity Pathway Analysis (IPA) software predicted the activation (*Z* > 2) of four biological function networks: D) proliferation of neuroglia, E,F) increased quantity and extension of cellular protrusions, and G) increased quantity of Ca^2+^.

To further explore the influence of millipascal shear stress on neural cell function, the genomic expression profile of VM cultures exposed to shear flow and static control conditions was analyzed using commercially available PCR arrays targeting 84 specific neurogenesis‐related genes and 84 genes belonging to the Hippo pathway family (**Figure** **4**A–G). The 18 most upregulated (Figure 4B) and 14 most downregulated (Figure 4C) genes were identified using a threshold value of *Z* > 2‐fold or *Z* < ‐2‐fold regulation (*Z* = flow vs static conditions).

The most significantly upregulated cell‐cycle associated gene in response to fluid shear stress was the apoptosis‐related gene caspase‐3 (Casp3) with a 102.54‐fold upregulation relative to VM populations cultured under static control conditions.^[^ ^69^ ^]^ Amyloid‐ß precursor protein (APP), associated with neuronal death also exhibited a 2.69‐fold upregulation in VM populations cultured under shear‐flow conditions.^[^ ^70^ ^]^ The two members of the S100 calcium‐binding protein family, A6 and B (S100a6 and S100b), involved in astrocyte proliferative processes, also underwent upregulations of 3.52‐ and 2.53‐fold, respectively.^[^ ^71^, ^72^ ^]^ Cyclin E1 and E2 (CCNE1 and CCNE2), reported to play a role in cell cycle and mitotic events, showed a positive regulation of 7.19‐ and 45.57‐fold, respectively.^[^ ^73^ ^]^ Similarly, casein kinase I isoform epsilon gene (CSNK1E), involved in DNA replication and repair, as well as circadian regulation exhibited upregulated expression of 9.19‐fold when compared to VM populations cultured under static control conditions.^[^ ^74^ ^]^

The most downregulated cell‐cycle associated gene induced by oscillatory fluid flow stimulation was hypoxanthine phosphoribosyltransferase 1 (HPRT1), with a 556.41‐fold downregulation, a gene linked to DNA and RNA production through purine recycling.^[^ ^75^ ^]^ Similarly, four‐jointed box kinase 1 (Fjx1) and glypican 5 (Gpc5), both involved in the control of cellular growth, division and differentiation were also downregulated by 2.51‐fold and 3.75‐fold, respectively.^[^ ^76^, ^77^ ^]^ Likewise, the phosphatase 2A catalytic subunit (PPP2CA) responsible for the negative control of cell proliferation and growth was also downregulated by 2.03‐fold.^[^ ^78^ ^]^

With respect to neurogenesis, the neuregulin 1 gene (NRG1) involved in growth and differentiation of both neurons and glial cells, displayed a 4.3‐fold upregulation in expression,^[^ ^79^ ^]^ along with the platelet‐activating factor acetylhydrolase IB subunit alpha (PAFAH1B1), a marker of neurogenesis, which exhibited a 3.81‐fold upregulation.^[^ ^80^ ^]^ Critically, neuronal differentiation 1 (Neurod1), a transcriptional activator responsible for neuronal morphogenesis and maintenance, underwent a 2.50 downregulation in expression.^[^ ^81^ ^]^ The expression of glial cell‐derived neurotrophic factor (GDNF), a promoter of neuron growth and survival underwent a 2.61‐fold upregulation.^[^ ^82^ ^]^ Concurrently, bone morphogenetic protein 4 (BMP4), also displayed a 2.42‐fold upregulation in expression, and has been shown to repress neurogenesis in vitro.^[^ ^83^ ^]^ Finally, angiomotin (AMOT) and angiomotin‐like 2 (AMOTL2), modulators of the Hippo signaling pathway, exhibited 7.31‐ and 3.97‐fold upregulations, respectively, in cells exposed to shear stimulation relative to cells cultured under static conditions.^[^ ^84^ ^]^

With respect to cell cytoskeleton and motility, the gene gamma‐actin (ACTG1) showed a 2.10‐fold upregulation.^[^ ^85^ ^]^ Similarly, doublecortin (DCX), a known cytoskeletal marker of neurogenesis exhibited a 4.3‐fold increase in expression.^[^ ^86^ ^]^ The ephrin B1 gene (EFNB1), which also plays a role in cell adhesion also displayed a positive regulation of 2.62‐fold.^[^ ^87^ ^]^ Conversely, roundabout guidance receptor (Robo1), an axon guidance, and cell adhesion receptor demonstrated a 2.10‐fold downregulation.^[^ ^88^ ^]^ Furthermore, the actin beta (ACTB) and hemicentin 1 (HMCN1), linked to cell structural integrity and motility along with adhesion and mechanotransduction, also demonstrated a 5.41‐fold and 2.27‐fold negative regulation in expression, respectively.^[^ ^89^, ^90^ ^]^ Interestingly, fluid‐shear stress also induced a 2.27‐fold downregulation of the casein kinase 1 delta gene (CSNK1D), indicative of Yap1 activation, a downstream actor of the “mechanosensing” Hippo signaling pathway.^[^ ^74^ ^]^ Moreover, the nephrocystin 4 gene (NPHP4) and protein tyrosine phosphatase non‐receptor type 14 (PTPN14), both act as negative regulators of the Hippo pathway by inhibiting TAZ and YAP and were downregulated by 2.27‐ and 2.54‐fold, respectively.^[^ ^91^, ^92^ ^]^

Next, Ingenuity Pathways Analysis (IPA) software was used to predict the activation or inhibition of biological functions, by creating functional networks using the differential gene expression between neural populations exposed to fluid shear or to static conditions. The IPA software predicted six significantly modified biological functions (activated *Z* > 2, inhibited Z <‐2), in VM cells after 14 days in culture following exposure to 6 h of 0.1 Pa of shear stress. Specifically, the upregulated expression of S100B, sonic hedgehog (SHH), superoxide dismutase 1 (SOD1), transforming growth factor beta 1 (TGFB1), C‐X‐C motif chemokine ligand 2 (CXCL2), erb‐b2 receptor kinase 2 (ERBB2), and GDNF together with downregulated expression of pleiotrophin (PTN) and Fibroblast growth factor 2 (FGF2) led to the predicted activation of “the proliferation of neuroglia” biological function (Figure 4D). In addition, grouping of the five positively modulated genes epidermal growth factor (EGF), mitogen‐activated protein kinase 10 (MAPK10), brain‐derived neurotrophic factor (BDNF), rac family small GTPase 1 (RAC1), and SHH predicted the activation of the “quantity of cellular protrusions” function (Figure 4E), along with the predicted activation of the “extension of cellular protrusions” function due to the upregulation of histone acetyltransferase p300 (EP300), netrin 1 (NTN1), PAFAH1B1, RAC1, apolipoprotein E (APOE), and the downregulation of reticulon 4 (RTN4) (Figure 4F). Moreover, the positive regulation of APP, APOE, B‐cell lymphoma 2 (BCL2), TGFB1, and EGF led to a predicted activation of the “Increased quantity of Ca^2+^” function (Figure 4G).

Interestingly, two related biological functions, despite a *Z*‐score under the threshold of significance, showed a *Z* > 1.5 predicting the activation of the “release of fatty acid” function (Figure S7A, Supporting Information) and a *Z* < ‐1.5, which predicts an inactivation of the “transport of monosaccharide” biological function (Figure S7B, Supporting Information).

Finally, the pathway identifier tool, comparing the transcriptome expression of neural populations exposed to 6 h of flow relative to static control conditions suggested a significant upregulation of the neuroinflammation pathway and biological functions related to proliferation and metabolism (Figure S7C, Supporting Information). Specifically, this pathway indicated a predicted activation of biological roles such as astrogliosis, neuronal damage, neurofibrillary tangles, Aß generation, apoptosis, oxidative stress, ROS production, BBB disruption, and Ca^2+^ overload.

### 2.5 Oscillatory Millipascal Fluid Shear Stress Promotes the Upregulation of Mechanosensitive Ion Channels

**Figure 5:**
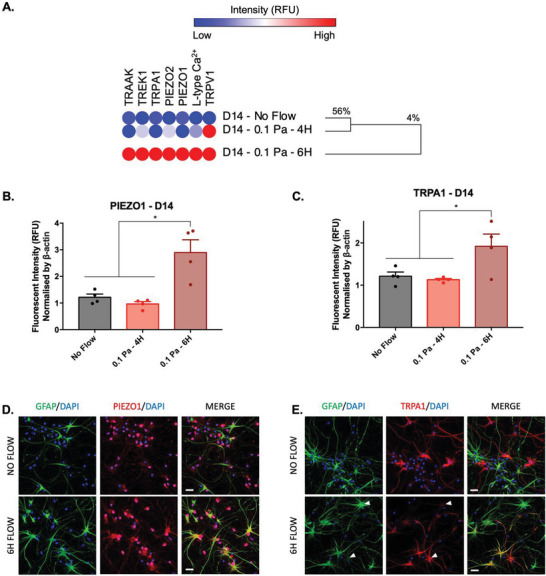
The mechanosensitive ion channel proteins PIEZO1 and TRPA1 are upregulated in response to millipascal fluid flow shear stress. A) A custom ion‐channel antibody microarray was used to generate a hierarchically clustered heatmap depicting binding intensities using complete linkage and Euclidean distances of scale normalized by ß‐actin (*n* = 4). Increased expression of all ion channels 14 days after exposure to 0.1 Pa shear flow for 6 h was observed. Quantitative analysis of antibody microarray fluorescence intensity of B) PIEZO1 and C) TRPA1 14 days after exposure to a 0.1 Pa shear flow for 6 h. These data were further confirmed using immunocytochemistry of D) DAPI (blue), GFAP (green), and PIEZO1 (red) or E) TRPA1 (red) (scale bar = 20 µm). Data are represented as mean ± SEM (*n* = 4). One‐way ANOVA with Tukey post hoc test was performed. * Represents a statistically significant difference of *p* < 0.05.

Transmembrane mechanosensitive ion channels such as members of the PIEZO or transient receptor potential (TRP) families have been shown to play a role in mediating cellular responses to physicomechanical stimuli through intracellular signaling cascades under both physiological and pathophysiological conditions.^[^ ^46^, ^93^ ^]^ To determine whether shear stress‐induced astrocyte reactivity was mediated by ion channel mechanoreception, an in‐house protein microarray was developed to assay the expression of known mechanically gated ion channels in primary VM populations subjected to millipascal shear stress relative to static control conditions. The two most upregulated receptors were subsequently further investigated in vitro using immunofluorescent imaging (**Figure** **5**).

Intriguingly, all tested mechanoreceptor proteins were upregulated following 4 h of 0.1 Pa flow shear stress, an effect which was exacerbated by increasing the stimulation time to 6 h (Figure 5A). Indeed, Euclidian cluster analysis using complete linkage returned two principal clusters 14 days poststimulation: Cluster 1 contained the 4 h shear flow group and the static conditions which exhibit 56% similarity and cluster 2, represented the 6 h flow condition and demonstrated 4% similarity with the 4 h shear flow group and the static condition cluster (Figure 5A).

Among the assayed ion channels, two members of the TRP cation channel family, the Vanilloid Receptor 1 (TRPV1) and Ankyrin 1 (TRPA1), which are known to be involved in mechanical, temperature, and chemical sensing, were upregulated following shear stress stimulation. In particular, TRPA1, which has been recognized to act as calcium influx regulator in astrocytes,^[^ ^94^, ^95^ ^]^ exhibited a significant 59% increase (control 1.21 vs treated 1.92; *p* = 0.0492) in expression after 6 h of 0.1 Pa shear stress, relative to cells cultured under static control conditions (Figure 5C).

Moreover, PIEZO1 and PIEZO2 were also overexpressed following 0.1 Pa oscillatory flow shear stress, with greater expression following 6 h of exposure (Figure 5A). Interestingly, PIEZO1 overexpression has been observed to occur in neurodegenerative disorders including Alzheimer's disease or multiple sclerosis.^[^ ^96^, ^97^ ^]^ In this study PIEZO1 displayed a significant ≈2.4‐fold increase in expression (control 1.22 vs treated 2.89; *p* = 0.0304) relative to cells cultured under static control conditions (Figure 5B).

PIEZO1 overexpression in VM populations exposed to fluid flow conditions was subsequently confirmed by immunocytochemistry which confirmed the presence of relatively large reactive astrocytes with a high GFAP intensity accompanied by a marked increase in PIEZO1 receptor staining intensity in both the astrocytic somas and cell ramifications (Figure 5D). Interestingly, upregulation of the TRPA1 receptor intensity appeared to negatively correlate with GFAP intensity in astrocyte populations (see white arrows, Figure 5E).

### 2.6 Neuroelectrode Implantation Triggers Astrocyte Overexpression of PIEZO1 and TRPA1 In Vivo

**Figure 6:**
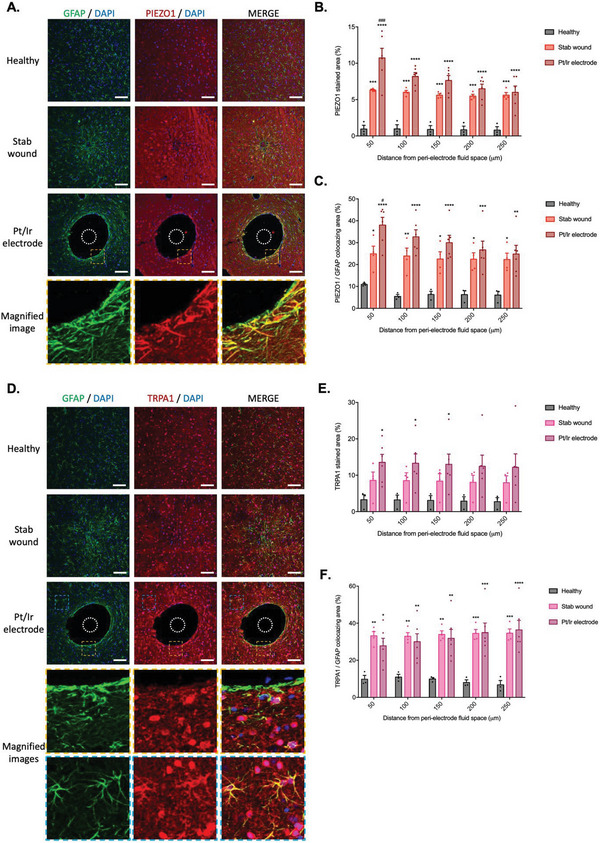
A) PIEZO1 and TRPA1 receptors are upregulated in vivo in peri‐electrode astrogliosis. To verify the prediction of the in vitro model, Pt/Ir electrodes were implanted in the rat subthalamic nucleus and either immediately removed (stab wound) or retained in situ for 8 weeks (Pt/Ir electrode). Representative immunohistochemistry images of nonimplanted healthy control tissue, an electrode stab injury, and Pt/Ir electrode‐implanted brain tissue, stained in green for GFAP, red for PIEZO1, and blue for DAPI, yellow dashed line depicts a zoomed region, the white dashed circle represents the electrode position (scale bar = 100 µm). B,C) Quantification of the PIEZO1 stained area (B) and colocalization of PIEZO1 to GFAP staining (C) relative to healthy control tissue. D) Representative immunohistochemistry images of non‐implanted healthy control tissue, an electrode stab injury, and Pt/Ir electrode‐implanted brain tissue, stained in green for GFAP, red for TRPA1, and blue for DAPI, yellow or blue dashed line depicts a zoomed region, the white dashed circle represents the electrode position (scale bar = 100 µm). Quantification of E) the TRPA1 stained area and F) colocalization of TRPA1 to GFAP staining relative to healthy control tissue. Data are represented as mean ± SEM (*n* = 3–6). Two‐way ANOVA with Tukey post hoc test was performed. *, **, ***, **** represent a statistically significant difference versus the healthy control and #, ##, ###, #### versus the stab wound condition (*p* < 0.05), (*p* < 0.01), (*p* < 0.001), and (*p* < 0.0001), respectively.

To explore further the relationship between neuroelectrode micromotion‐induced fluid shear stress on astrocyte reactivity and the expression of PIEZO1 and TRPA1, we studied peri‐electrode glial scarring with implanted commercially available neuroelectrodes in the rat subthalamic nuclei. Probes were either removed immediately after insertion to create a stab wound or anchored to the skull (establishing relative micromotion at the tissue/electrode interface due to animal respiration) and retained in place for 8 weeks to generate a neuroelectrode‐induced glial scar (**Figure** **6**; Figure S8, Supporting Information).

Notably, a peri‐electrode void of ≈4 times the electrode diameter (389 ± 71 µm in comparison to the 100 µm cross‐section of the SNEX‐100 electrode tip) was observed only in the in situ implantation group (Figure S8B, Supporting Information), a phenomenon reported previously.^[^ ^18^, ^19^, ^24^, ^25^, ^65^ ^]^ We next assessed astrocyte and microglia aggregation and neuronal loss (well described indicators of gliosis)^[^ ^1^, ^6^ ^]^ at the peri‐electrode region to validate the robustness of our model (Figure S8C, Supporting Information). Indeed, in both the stab injury and neuroelectrode insertion conditions, the GFAP stained area exhibited a significant increase of ≈70% and ≈80%, respectively (*p* = 0.0004 and p < 0.0001) at a distance of 50 µm from the insertion site, and an increase of ≈56% and ≈59% (n.s. and *p* = 0.0165), respectively, at a distance of 250 µm from the insertion site relative to healthy control tissues (Figure S8D, Supporting Information). Similarly, electrode insertion led to an ≈63% increase in peri‐electrode Iba1 staining area in the stab wound group and ≈78% increase in stained area in the Pt/Ir electrode in situ group (n.s. and *p* = 0.0001, respectively) at a distance of 50 µm from the implantation site, relative to the healthy tissue control group. This effect was sustained up to a distance of 150 µm from the peri‐implant fluid space and a significant ≈65% increase in microglia staining area (*p* = 0.0499) was noted in animals implanted with the platinum electrode in situ (Figure S8E, Supporting Information).

Conversely, a ≈96% (*p* < 0.0001) decrease in peri‐electrode neuron density was observed in the stab injury group and a significant decrease of ≈99% (*p* < 0.0001) in the in situ Pt/Ir electrode group at a distance of 50 µm from the peri‐electrode fluid space, relative to healthy control tissues. Neuronal loss was also present at a distance of 250 µm from the peri‐electrode fluid space and significant reductions in neuron density of ≈62% and 72% (*p* < 0.0001 and *p* < 0.0001) were observed in both the stab injury and Pt/Ir electrode groups, respectively (Figure S8F, Supporting Information).

The expression of the mechanosensitive ion channels PIEZO1 and TRPA1 in both the stab wound and the in situ peri‐electrode glia scar was also assessed in vivo relative to healthy control tissue via analysis of staining area and the colocalization of PIEZO1 and TRPA1 with the astrocytic marker GFAP (Figure 6A,D). Both in situ electrode and stab wound groups presented a marked and significant increase in staining area of PIEZO1, specifically, an increase of ≈85% (*p* = 0.0001) for the stab injury and ≈91% (*p* < 0.0001) for the in situ electrode group was observed at a distance of 50 µm from the peri‐electrode fluid space, which persisted as an ≈86% increase in staining area (*p* = 0.0005 and *p* < 0.0001) at a distance of 250 µm from the implantation site, for both experimental groups (Figure 6B).

Notably, PIEZO1/GFAP colocalization was observed to follow a similar trend when compared to healthy control tissues, and significantly greater colocalization was measured up to a distance of 250 µm from the peri‐electrode fluid space in both experimental groups. PIEZO1/GFAP colocalization was observed to increase from ≈56% (*p* = 0.0392) for the stab wound and ≈71% (*p* < 0.0001) for the electrode group, at a distance of 50 µm from the electrode interface, to ≈72% (*p* = 0.0140) and ≈75% (*p* = 0.0017), respectively, at a distance of 250 µm from the peri‐electrode fluid space (Figure 6C).

Similarly, a significant ≈75% increase in the total stained area of TRPA1 was observed in the Pt/Ir electrode in situ group at a distance of 50 µm (*p* = 0.0341), 100 µm (*p* = 0.0374), and 150 µm (*p* = 0.0418) from the insertion site relative to control healthy tissues (Figure 6E). Interestingly, TRPA1/GFAP colocalization demonstrated a consistent increase as a function of distance in both experimental groups, with a ≈70% (*p* = 0.0023) and ≈65% (*p* = 0.0123) colocalization increase respectively within the first 50 µm, which increased to ≈80% (*p* = 0.0003 and *p* < 0.0001, respectively) colocalization 250 µm from the implantation site (Figure 6F).

### 2.7 PIEZO1 Inhibition Promotes Astrocyte Reactivity and Neuronal Loss, While PIEZO1 Overactivation Decreases Astrocyte Reactivity and Increases Neuron Viability In Vitro

**Figure 7:**
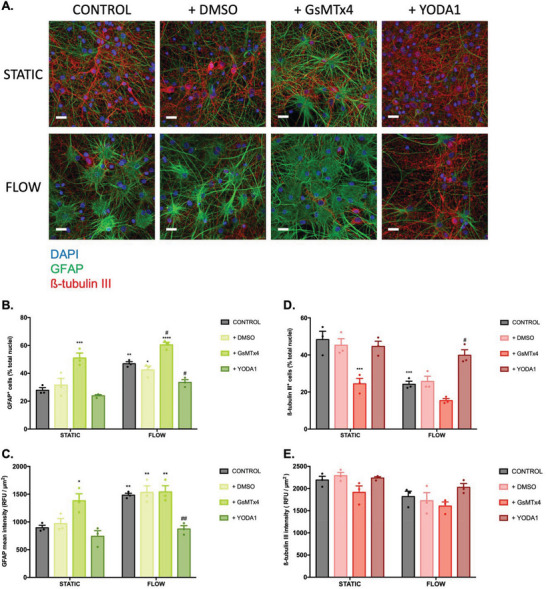
A) PIEZO1 inhibition promotes astrocyte reactivity and neurodegeneration while PIEZO1 activation rescues astrocyte reactivity triggered by shear stress. To evaluate the action of the PIEZO1 chemical agonist Yoda1 and the PIEZO1 antagonist GsMTx4 on astrocyte reactivity and neuron viability, VM populations were exposed to flow shear stress (0.1 Pa, 6 h) and supplemented with 10 µm of Yoda1/DMSO or 0.5 µm of GsMTx4 for 14 days and stained for GFAP and ß‐tubulin III (A) (scale bar = 20 µm; *n* = 3). B) Quantitative analysis of GFAP positive cell number in response to Yoda1, GsMTx4, and control DMSO under flow and static culture conditions. C) Quantification of GFAP intensity in astrocyte populations in response to Yoda1, GsMTx4, and control DMSO under flow and static culture conditions. D) Quantitative analysis of ß‐tubulin III positive cell number in response to Yoda1, GsMTx4, and control DMSO under flow and static culture conditions. E) Quantitative analysis of ß‐tubulin III intensity of VM cells in response to Yoda1, GsMTx4, and control DMSO under flow and static culture conditions. Data are represented as mean ± SEM (*n* = 3–4). Two‐way ANOVA with Tukey post hoc test was performed. *, **, ***, **** represent a statistically significant difference versus the static control and #, ##, ###, #### versus the flow control (*p* < 0.05), (*p* < 0.01), (*p* < 0.001), and (*p* < 0.0001), respectively.

To further understand the role played by PIEZO1 in the development of peri‐electrode gliosis and to explore the potential for ion channel inhibitors or activators in antigliosis therapy, static and dynamic (6 h, 0.1 Pa) VM cell cultures were supplemented with GsMTx4 and Yoda1, established chemical antagonists and agonists of PIEZO1, respectively, for 14 days. Astrocyte and neuron density were subsequently assessed by fluorescent labeling of GFAP and *β*‐tubulin III, respectively (**Figure** **7**). Both inhibition and activation of PIEZO1 significantly modulated astrocyte and neuron density relative to control culture conditions, effects which were further exacerbated in VM populations exposed to flow shear stress conditions (Figure 7A).

Quantification of GFAP^+^ cells in a mixed VM culture under static culture conditions revealed a significant ≈45% increase in astrocyte number (*p* = 0.0003) following exposure to the PIEZO1 inhibitor GsMTx4, which was similar to the observed increase in astrocyte number resulting from culture under oscillatory fluid flow only (*p* = 0.0022). Furthermore, combining fluid shear stress stimulation with inhibition of PIEZO1 through GsMTx4 exposure further amplified astrocyte proliferation, indicated by a significant ≈22% (*p* = 0.0382) increase in GFAP^+^ cell density relative to VM cells cultured under fluid flow alone and by a significant ≈54% (*p* < 0.0001) increase in GFAP^+^ cell density relative to VM cells cultured under static control conditions. Interestingly, activation of PIEZO1 using the chemical agonist Yoda1, inhibited the proliferative effects of fluid shear stress on astrocyte cells and significantly reduced astrocyte density by ≈29% (*p* = 0.0395) relative to cells cultured under fluid flow conditions alone, indeed no significant differences were observed in astrocyte cell density in VM populations exposed to Yoda supplementation under fluid flow conditions relative to VM populations cultured under static control conditions (Figure 7B).

Similarly, image analysis revealed a significant ≈35% increase (*p* = 0.0298) in mean GFAP fluorescence intensity in VM cells exposed to the PIEZO1 inhibitor GsMTx4 when cultured under static conditions relative to cells cultured under static control conditions alone. GFAP intensity was observed to increase by ≈40% (*p* = 0.0065) in VM cells cultured with GsMTx4 supplementation and under oscillatory flow conditions relative to VM cells cultured under static control conditions alone. Conversely, Yoda1 supplementation of VM cells cultured under fluid flow conditions significantly decreased astrocyte GFAP fluorescence intensity by ≈41% (*p* = 0.0046) relative to VM cells cultured under fluid flow conditions alone, returning the mean GFAP intensity to that of astrocytes cultured under static control conditions (Figure 7C).

In a similar manner, the number of *β*‐tubulin III^+^ cells was quantified in response to experimental flow conditions and the chemical induction of PIEZO1 inhibition/activation. Here, the addition of GsMTx4 to the static cultures led to a significant ≈49% reduction (*p* = 0.0006) in the neuronal population density relative to cells cultured under static control conditions, comparable to VM cells cultured under fluid shear stress conditions alone. Critically, exposure to the PIEZO1 antagonist GsMTx4 induced a further (insignificant) ≈20% decrease in the density of *β*‐tubulin III positive cells relative to cells cultured under experimental flow conditions alone.

Conversely, exposure to the PIEZO1 agonist Yoda1 significantly protected against shear stress mediated neuron loss, inducing an ≈39% (*p* = 0.0289) increase in the number of *β*‐tubulin III positive cells relative to cells cultured under fluid flow conditions alone, resulting in a comparable neuron density to that of VM cells cultured under static control conditions (Figure 7D). Similarly, analysis of *β*‐tubulin III indicated an ≈13% (n.s.) reduction in mean fluorescence intensity in VM neurons cultured in media supplemented with GsMTx4 and under static conditions and an ≈12% (n.s.) reduction in mean fluorescence intensity in VM neurons cultured in media supplemented with GsMTx4 and under fluid flow conditions, relative to cells cultured under static conditions alone and under flow conditions alone, respectively (Figure 7E).

### 2.8 PIEZO1 Inhibition of Neural Populations Reduces Mitochondrial Oxygen Consumption Rate and Extracellular Acidification Rate through Glycolysis Impairment

**Figure 8:**
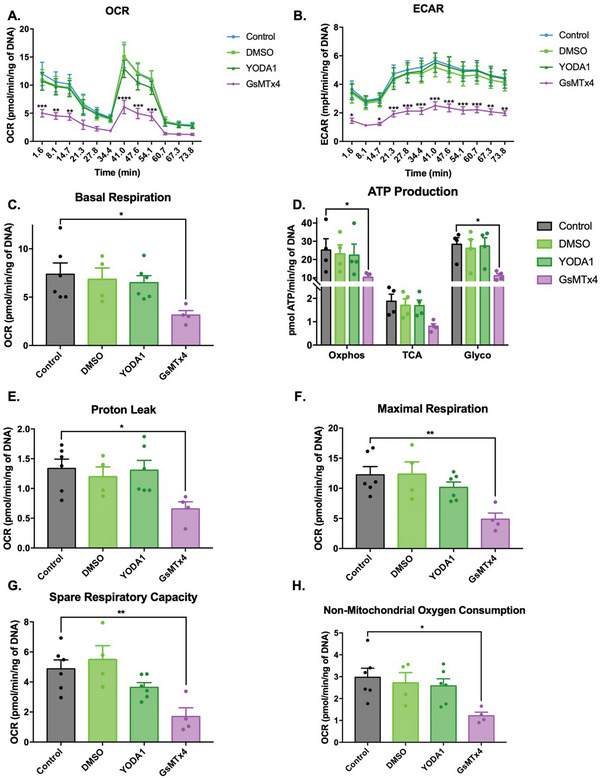
PIEZO1 inhibition affects the oxygen consumption and extracellular acidification rate in VM populations through impairment of glycolysis processes. To understand the mechanism of action through which GsMTx4 and Yoda1 modulate astrocyte activation, an extracellular flux analysis investigating the mitochondrial health state was performed using the MitoStress Test. Analysis of the effect of PIEZO1 activation and inhibition on A) cellular oxygen consumption (OCR) and B) extracellular acidification rate (ECAR) relative to cells cultured under control conditions. Analysis of the effect of C) PIEZO1 activation and inhibition on basal respiration, D) ATP production, E) proton leak processes, F) maximal respiration, G) the spare respiratory capacity, and H) non‐mitochondrial oxygen consumption relative to VM populations cultured under DMSO alone or control conditions. Data are represented as mean ± SEM (*n* = 4–6). Two‐way ANOVA with Tukey post hoc test was performed. *, **, ***, **** represent a statistically significant difference versus control (*p* < 0.05), (*p* < 0.01), (*p* < 0.001), and (*p* < 0.0001), respectively.

To probe the impact of PIEZO1 modulation on neural metabolic activity, we assessed the effects of its chemical activation and inhibition on mitochondrial oxygen consumption rate (OCR) and extracellular acidification rate (ECAR) in vitro. Naïve VM cells were exposed to the PIEZO1 agonist Yoda1 and antagonist GsMTx4 for 14 days in culture prior to extracellular flux analysis (Figure S9A, Supporting Information). Overall, it was observed that GsMTx4 supplementation negatively affected both OCR and ECAR suggesting an effect on respiration and glycolysis. Conversely, Yoda1 supplementation did not significantly modify OCR or ECAR in VM populations (**Figure** **8**A,B).

Specifically, inhibition of PIEZO1 by GsMTx4 led to a significant ≈57% (*p* = 0.0364) reduction in mitochondrial basal respiration relative to cells cultured under control conditions, while activation by Yoda1 did not modulate basal respiration (Figure 8C). Remarkably, GsMTx4 exposure significantly reduced ATP productions in VM cells. In particular, the oxidative phosphorylation pathway (Oxphos), the Krebs cycle (TCA), and the glycolysis cycle (Glyco) were all significantly downregulated by ≈59% (*p* = 0.0324), ≈56% (n.s.), and ≈61% (*p* = 0.011), respectively, relative to VM cells cultured under control conditions (Figure 8D). Similarly, maximal respiration was significantly impaired by PIEZO1 inhibition inducing a significant ≈60% (*p* = 0.0073) reduction relative to VM cells cultured under control conditions (Figure 8F). The spare respiratory capacity was also significantly modulated by PIEZO1 inhibition with a decrease of ≈65% (*p* = 0.0091) relative to VM cells cultured under control conditions (Figure 8G). Interestingly, the non‐mitochondrial oxygen consumption was also affected in VM populations exposed to the PIEZO1 antagonist, revealing a significant reduction of ≈59% (*p* = 0.0227) relative to VM cells cultured under control conditions (Figure 8H). PIEZO1 inhibition also induced a significant ≈50% (*p* = 0.04) decrease in mitochondrial proton leak relative to VM cells cultured under control conditions (Figure 8E). However, it was noted that neither DNA content nor the mitochondria bioenergetic health index was significantly modulated in any of the control or experimental groups, implying the principal reduction in mitochondria function was independent of cell loss or disruption to cellular health processes (Figure S9D,E, Supporting Information). Collectively, these findings suggest that the induction of a reactive astrocyte phenotype via GsMTx4 inhibition of PIEZO1 is accompanied by glycolysis impairment and diminished ATP production.

## 3 Discussion

**Figure 9:**
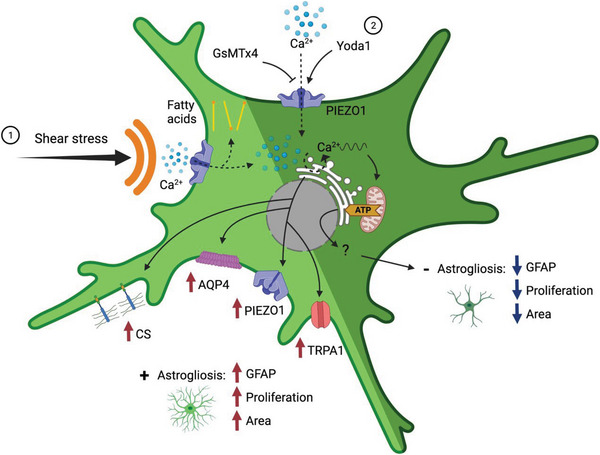
Proposed mechanisms of shear stress‐mediated astrocyte activation and PIEZO1 inhibition with GsMTx4 or its overactivation with Yoda1 leading to the exacerbation or rescue of astrogliosis. (1) Shear stress applied to astrocytes, either through in vitro oscillatory fluid flow stimulation or in vivo electrode micromotion, opens mechanosensitive ion channels which facilitate calcium ion flux, triggering the overexpression of the glial fibrillary acid protein (GFAP), chondroitin sulfates (CS), aquaporin‐4 (AQP4), piezo‐type mechanosensitive ion channel component 1 (PIEZO1), transient receptor ankyrin 1 (TRPA1), and leading to an increase in cell proliferation. (2) Chemical inhibition of PIEZO1 with GsMTx4 reduces calcium flux and the activation of associated calcium signalling pathways, which leads to an overall reduction in mitochondria‐mediated glycolysis and ATP production, resulting in increased astrocyte reactivity and reduced astrocyte‐mediated neuronal support functions. Conversely, overactivation of the PIEZO1 channel using the agonist Yoda1 results in increased Ca^2+^ influx initiating ER‐stored calcium waves, which induces a rise of mitochondrial metabolic and energetic functions resulting in reduced astrocyte reactivity. Schematic created with BioRender.com.

In this work, we provide evidence for the role of sub‐Pascal shear stress in promoting reactive gliosis, and the evolution of the fluid‐filled space at the peri‐electrode interface and propose a mechanism of action mediated by the mechanical activation of transmembrane ion channels in astrocyte populations (**Figure** **9**). Specifically, we developed an in silico model and show that respiration‐mediated electrode micromotion is translated into milliscale shear stress and propagated through the peri‐electrode fluid‐filled space to the receding neural tissues (Figure 1). Notably, we show that the shear stress amplitude is inversely proportional to the electrode–tissue distance and suggest that the peri‐electrode fluid space reaches a steady state when a tissue shear stress of <0.1 Pa is achieved through tissue regression. We went on develop an in silico informed in vitro model of neuroelectrode micromotion‐induced fluid shear stress derived from a parallel‐plate flow chamber. We subsequently investigated the effects of physiologically relevant shear stress on multiple indicators of astrocyte reactivity, inflammation, and neurogenesis (Figures 2, 3, 4). Critically, we identified that PIEZO1 and TRPA1 became significantly upregulated in vitro in response to fluid shear stress and went on to confirm these upregulations in the peri‐implant tissues using an in vivo model of gliosis (Figures 5 and 6). Finally, we demonstrated that chemical activation of PIEZO1 attenuated the expression of biochemical markers of gliosis and stimulated neuronal regeneration in vitro. Conversely, inhibition of PIEZO1 promoted a reactive astrocyte phenotype (Figure 7). Finally, we showed that PIEZO1 inhibition impaired mitochondrial functions including ATP production, respiration, and glycolysis in vitro (Figure 8).

Previous in vivo studies have demonstrated conclusively that micromotion‐induced peri‐electrode shear stress contributes significantly to the development of glial scarring.^[^ ^24^, ^25^, ^65^ ^]^ Furthermore, a dynamic process of tissue regression from the electrode implantation site and a reduction in device integration is frequently observed weeks to months following implantation.^[^ ^1^ ^]^ This tissue recession leads to the development of a radially expanding peri‐electrode interstitial fluid‐filled space, which generally reaches a steady state at 3–4 times the diameter of the implanted device^[^ ^18^, ^19^, ^24^, ^25^, ^65^ ^]^ and is filled with an interstitial fluid composed of an inhomogeneous mix of cerebrospinal fluid, cell debris, and extracellular matrix, with an average viscosity of ≈30 mPa s.^[^ ^66^ ^]^ Furthermore, this growing extracellular space has been shown to be exacerbated in devices fixed to the skull^[^ ^24^, ^25^ ^]^ due to an increase in relative micromotion magnitude.^[^ ^30^, ^65^ ^]^

It can be hypothesized that the fluid within the peri‐electrode void undergoes localized flow in response to electrode micromotion, imparting shear stress onto the peri‐electrode tissues, leading to cell death and void expansion before reaching a steady state electrode–tissue distance, at which shear stresses no longer induce necrosis but promote a state of chronic astrocyte reactivity, leading to glial scar development. Critically, we show that the evolution of this space occurs in the absence of electrical stimulation.

The underlying molecular mechanisms of peri‐implant gliosis remains unclear^[^ ^1^ ^]^ and research has failed to develop comprehensive in vitro models which recapitulate the complex glial scarring process.^[^ ^98^ ^]^ Most current in vitro models of gliosis induce glial cell proliferation and activation, as well as trigger neurodegeneration, however they do not elicit the expression of concurrent or chronic markers of glial scar development.^[^ ^57^, ^58^, ^59^, ^98^ ^]^ In this study, a physiologically representative oscillatory fluid flow shear stress model was found to trigger astrogliosis and neuronal loss, coupled with an overexpression of CS and a downregulation of the glypican 5 gene. This is relevant as in vivo studies of glial scar tissue have revealed a disbalance of heparan/chondroitin sulfates distribution, which inhibit neuronal growth and axonal regeneration as well as perpetuate inflammation.^[^ ^5^, ^99^, ^100^ ^]^ Furthermore, the fluid shear stress model induced an overexpression of the water channel AQP4 in VM astrocytes, an established marker of astrocyte reactivity and initiator of fibrosis through perturbation of transcellular water transport mechanisms.^[^ ^68^, ^101^ ^]^

The effects of in vitro shear stress on the proinflammatory state of VM cell populations were further validated via protein microarray analysis and modulation to GFAP and ß‐tubulin III expression, as well as decreased Myelin synthesis, indicating a loss of neuron and axon integrity were noted.^[^ ^102^, ^103^ ^]^ Moreover, proteomic analysis also revealed increases in the synthesis of established brain injury/inflammation markers, including Nestin,^[^ ^104^, ^105^ ^]^ Olig2,^[^ ^106^, ^107^ ^]^ CD81,^[^ ^108^, ^109^ ^]^ and OX42.^[^ ^110^, ^111^ ^]^

In vitro fluid flow shear stress was also shown to initiate genomic changes resembling those reported in brain injury and neuroinflammatory contexts. Specifically, the upregulation of Casp3 and App, coupled with a simultaneous decrease in the expression of ACTB, depicts the onset of the neuronal apoptosis,^[^ ^69^, ^70^, ^89^ ^]^ which was further indicated by the downregulation of neurogenesis‐related genes Neurod1, Robo1, tyrosine 3‐monooxygenase activation protein theta (Ywhaq), and HMCN1.^[^ ^81^, ^88^, ^90^, ^112^ ^]^ Furthermore, the increased expression of two astrocytosis specific genes, S100a6 and S100B, points to a proliferative state of the astroglial population,^[^ ^71^, ^72^ ^]^ further asserted by the positive regulation of markers of cell development, differentiation, proliferation, and cycle regulation CCN1, CCN2, CSNK1E, ACTG1, EFNB1, crumb cell polarity complex component 2 and 3 (CRB2 and CRB3), which are also reported as associated with glioblastoma, brain injury, and astrocyte reactivity.^[^ ^73^, ^87^, ^113^ ^]^

Interestingly, VM cultures exposed to flow shear stress conditions also exhibited upregulated expression of NRG1, PAFAH1B1, DCX, and GDNF and downregulation of necdin (NDN), which suggest a protective and/or survival response triggered by the neuronal population in reaction to increased proliferation of astrocytes.^[^ ^79^, ^80^, ^82^, ^86^ ^]^ Conversely, the upregulation of BMP4 has been shown to act as a suppressor of neurogenesis and to play a role in sustaining astrogenesis,^[^ ^83^, ^114^ ^]^ which suggest a retrocontrol of this neuronal‐rescue process. Interestingly, homeodomain interacting protein kinase 2 (HIPK2) also exhibited downregulated expression, which has been shown to occur in response to noxious stimuli (such as shear stress), to alter tumor protein p53 expression and lead to neuron dysfunction.^[^ ^115^ ^]^ In addition, the decreased expression of NPHP4 and PTPN14, suggests inhibition of the Yap/Taz complex and an activation of the Hippo signaling pathway.^[^ ^91^, ^92^ ^]^

Interestingly, our in vitro model did not contain microglia or oligodendrocytes (due to low numbers in the midbrain at this developmental stage),^[^ ^116^, ^117^ ^]^ suggesting that the neurodegeneration observed from 7 day poststimulation was derived from an increased astrocyte reactivity. In fact, Liddelow and Barres proposed a mechanism where astrocytes release neurotoxic factors following injury,^[^ ^118^ ^]^ recently described as saturated lipids.^[^ ^119^ ^]^ This hypothesis correlates with our genomic data analysis, which also predicted the release of fatty acids from VM populations following exposure to shear stress in vitro (Figure S7B, Supporting Information).

Mechanically gated ion channels have recently been implicated as playing key roles in the homeostasis of the CNS and in mediating mechanotransduction in neural populations.^[^ ^32^, ^33^, ^34^, ^35^, ^36^, ^37^, ^38^, ^39^, ^120^ ^]^ Indeed, several ion channels have been shown to play a role in the reception of mechanical cues and have been shown to become perturbed in the progression of multiple disease states.^[^ ^17^, ^46^, ^121^ ^]^ In particular astrocytic PIEZO1 has been shown to be overexpressed in tissues with elevated beta‐amyloid deposition and in glioma masses due to increased tissue rigidity.^[^ ^47^, ^54^ ^]^ TRPA1 has also been described as upregulated in astrocytes and Schwann cells following neural inflammation and injury.^[^ ^38^ ^]^ Moreover, both TRPA1 and PIEZO1 have been implicated as mediators of the astrocyte neuroinflammatory response by modulating the synthesis of proinflammatory cytokines^[^ ^41^, ^122^ ^]^ or hormones.^[^ ^42^ ^]^

Here, PIEZO1 overexpression in reactive astrocyte populations (as confirmed by elevated GFAP synthesis) was observed in vitro and at the peri‐electrode site in vivo, indicating that these structures may play a role in mechanosensation of fluid flow‐generated shear stress. It can further be hypothesized that PIEZO1 overexpression in astrocyte populations induces a cellular shift toward a proinflammatory phenotype, as demonstrated in macrophages by Atcha et al.^[^ ^123^ ^]^ and can play a role in establishing chronic peri‐electrode inflammation.^[^ ^121^ ^]^

Interestingly, in both in vitro and in vivo models, TRPA1 displayed a more complex expression profile; indeed, the TRPA1 receptor was upregulated in VM populations exposed to shear stress conditions, however, its presence was more abundant in astrocytes populations with a moderate GFAP content and spatially more distant from the vicinity of the electrode. This expression profile may due to the role of astrocytic TRPA1 in calcium‐induced glutamate release which has been shown to activate neighboring neurons.^[^ ^94^, ^95^, ^124^, ^125^ ^]^ It can be hypothesized that TRPA1 expression is localized to those astrocyte populations, which interact with neuronal cells and which are less abundant at the peri‐electrode interface.

To better understand the role of MS ion channels, in both physiological and pathological contexts and to assess their potential as therapeutic targets, chemical inhibitor, GsMTx4, and activator Yoda1 can be employed to modulated PIEZO1 channel activation in vitro.^[^ ^34^, ^35^, ^41^, ^47^, ^126^, ^127^, ^128^ ^]^ Here it was observed that GsMTx4‐induced PIEZO1 inhibition led to astrocyte reactivity and neurodegeneration and potentiated the proinflammatory effects of fluid flow shear stress in vitro. Pathak et al. showed comparable results in neural stem cells, and both chemical or siRNA‐mediated PIEZO1 inhibition stimulated astrogenesis and reduced neurogenesis through a decrease in Yap mediated nucleotranslocation.^[^ ^34^ ^]^ Moreover, Liu et al. revealed that the inhibition of PIEZO1 increases the release of pro‐inflammatory mediators in microglia,^[^ ^129^ ^]^ again suggesting a progliosis effect of PIEZO1 inhibition.

Conversely, PIEZO1 activation through Yoda1 exposure was observed to negate the proinflammatory effects triggered by oscillatory fluid shear stress. This is in agreement with previous studies by Velasco‐Estevez et al. and Malko et al., who showed that the activation of PIEZO1 in LPS exposed cortical astrocytes and primary microglia led to a reduction in the synthesis of proinflammatory cytokines and reduced gliosis in vitro.^[^ ^41^, ^122^ ^]^ Several further studies have also showed that PIEZO1 is upregulated after axon/neuronal injury and that its loss or inhibition boost axonogenesis and regeneration,^[^ ^35^, ^130^, ^131^, ^132^ ^]^ suggesting multiple and contradictory roles for the same mechanosensitive ion channel PIEZO1 according to the cell type and to physiological context.

In this study, it was observed in vitro that fluid shear stress induced an upregulation in PIEZO1 expression, in conjunction with astrocyte activation and neural degeneration. Conversely, chemical activation of PIEZO1 was observed to attenuate astrocyte reactivity. This seemingly contradictory effect may be explained by considering that shear stresses may activate multiple ion channel types (such as TRPA1), which together can act on diverse signalization pathways, while chemical activation via PIEZO1 is specific. Additionally, a rescue mechanism can be proposed, whereby increased astrocytic PIEZO1 expression aids to raise the level of PIEZO1 activation in order to attenuate the on‐going reactivity process.

Interestingly, a recent study by Swain et al. has shown that PIEZO1 activation through either shear stress or Yoda1 exposure, leads to increased synthesis of PLA2 in pancreatic cells, an enzyme involved in fatty acid release (an effect predicted by our genomic data), which triggers the opening of TRPV4 channels and perpetrates dysregulated calcium flux, resulting in mitochondria dysfunction.^[^ ^133^ ^]^ Subsequently, a growing body of research has shown that restoration of astrocytes and neural cell metabolism following CNS traumatic injury or disease, can produce a neuroprotective effect and reduce coinciding astrogliosis.^[^ ^134^, ^135^, ^136^, ^137^ ^]^

Recently, several studies have linked mechanosensing^[^ ^138^ ^]^ MS ion channels,^[^ ^139^ ^]^ and particularly the activation of PIEZO1 with an increased release of ATP.^[^ ^126^, ^140^, ^141^, ^142^, ^143^ ^]^ To investigate the role of PIEZO1 in regulating neural metabolism in vitro, we combined metabolic flux analysis with PIEZO1 chemical modulation and observed that, GsMTx4‐induced PIEZO1 inhibition increased proinflammatory process and decreased critical mitochondrial functions in VM cell populations, including glycolysis and ATP production, effects which were not observed with Yoda1‐mediated PIEZO1 activation. Interestingly, Lopez‐Fabuel et al. showed that decreased mitochondrial function and increased ROS production, promotes astrocyte survival while negatively influencing neuron viability.^[^ ^144^ ^]^ This effect was further described by Weber and Barros, who discussed the “selfishness” of reactive astrocytes, observing that following injury or in response to disease, activated astrocytes will alter their metabolism and reduced their neuronal support functions to favor their own survival.^[^ ^145^, ^146^ ^]^

Together, these data indicate that PIEZO1 activation impacts mitochondrial functions and cellular metabolism, which are crucial for the homeostasis of neural tissues^[^ ^147^, ^148^ ^]^ and play an important role in controlling both neuron and astrocyte functions.^[^ ^34^ ^]^ Mechanistically, it can be hypothesized that shear stress‐induced MS channel activation promotes a sustained increase in intracellular calcium transportation processes, which leads to the release of fatty acids and to the impairment of mitochondrial functions, drastically reducing ATP production and promoting neuronal death and astrocyte reactivity (Figure 9).

In conclusion, we have shown that neuroelectrode micromotion is translated into milliscale shear stress at the tissue–electrode interface and hypothesize that this mechanism gives rise to the peri‐electrode fluid filled space. Our work suggests that the mechanosensitive ion channels PIEZO1 and TRPA1 are key mediators of peri‐electrode astrogliosis and act as regulators of critical metabolic process in neural populations. Finally, it may be inferred that electrode functionalization with chemical agonist/antagonist of PIEZO1 may promote chronic electrode stability in vivo.

## 4 Experimental Section

### Computational Modeling of Neuroelectrode Micromotion within the Filled Peri‐Electrode Fluid Space

A FVM reproducing WSS at the electrode/peri‐electrode fluid space/brain tissue interface was developed using ANSYS Workbench 2021 (ANSYS, Inc., Canonsburg, PA, USA). A 2D parametric model of a neuroelectrode device (based on SNEX‐100 electrode, Microprobes for Life Science, Gaithersburg, USA) featuring a cylindrical stepped tip was developed in SpaceClaim. The peri‐electrode region filled with brain interstitial fluid was represented by a cavity between the electrode and the brain tissue (Figure 1). The generated quadrilateral numerical mesh of the fluid domain consisted of 1376 cells and 1557 nodes (physics preference – CFD; solver preference – Fluent) and was validated in the Fluent/Setup module. The brain interstitial fluid density was assumed to be 1006 kg m^−3^ (based on cerebrospinal fluid density),^[^ ^149^ ^]^ and a viscosity range from 1.2 to 100 mPa s was selected based on previous work.^[^ ^66^, ^150^ ^]^ The electrode tip was assigned material properties of stainless steel 316L, austenitic, AISI 316L, according to data compiled by Ansys Granta. The mechanical properties of brain tissues were extracted from the literature (density 1060 kg m^−3^, Young's modulus 6 kPa, Poisson's ratio 0.45).^[^ ^60^, ^61^ ^]^ The temperature was kept constant at 37 °C.

Transport equations were based on the viscous SST *k*–*ω* turbulence model and electrode micromotion was simulated by the displacement of the probe along the *x*‐axis while the tissue remained relatively fixed. Based on previously published animal model experimental results, a micromotion amplitude of 15 µm (30 µm peak‐to‐peak) was applied with a frequency of 0.5 Hz arising from respiration.^[^ ^29^, ^151^, ^152^ ^]^ The WSS was calculated as an average value perpendicular to the direction of electrode oscillation. The calculated WSS value was then used to inform the flow parameters of the in silico and in vitro PPFC studies.

### Numerical 3D‐Simulation of Oscillatory Wall Shear Stress in the Parallel Plate Flow Chamber

To determine the consistency of fluid flow forces on a cell monolayer, CFD characterization of the developed PPFC was performed using ANSYS Fluent (Figure S1, Supporting Information). A 3D model was reconstructed in SpaceClaim from technical sheets provided by the PPFC's producer (Department of Mechanical and Manufacturing Engineering, School of Engineering, Trinity College Dublin, Ireland), which was further verified with chamber dimension measurements using both digital electronic depth gauge and Vernier calliper. The generated numerical mesh consisted of 794 859 nodes and 426 604 elements (physics preference, CFD; solver preference, Fluent). The properties of the polycarbonate material used for the PPFC construction were based on the Fluent Solid Materials database (density 1200 kg m^−3^, specific heat 1250 J °C^−1^ kg^−1^, thermal conductivity 0.2 W °C^−1^ m^−1^. The fluid domain consisted of Dulbecco's modified Eagle's medium/F12 (DMEM/F12)–1% fetal bovine serum (FBS) solution (density 1009 kg m^−3^, dynamic viscosity 0.930 mPa s).^[^ ^153^ ^]^ A transient analysis of the viscous model at 37 °C was carried out to simulate the oscillatory fluid flow in the microchannel with a frequency of 0.5 Hz as a result of the change of the flow direction at the PPFC inlet. A volumetric flow rate in the range of 4.49 to 381 mL min^−1^ (Table S1, Supporting Information) was applied.

### Animal Ethical Statement

Research and animal procedures were performed in accordance with the European (EU) guidelines (2010/63/UE) and Health Products Regulatory Authority of Ireland. Every effort was made to minimize animal suffering and to reduce the number of animals used.

### Ventral Mesencephalic Primary Cell Culture from E14 Rat Embryos

The mesencephalon is a well‐established implantation site for deep brain stimulation electrodes. In order to assess the in vitro response of a clinically representative subpopulation of CNS neurons, VM cells were extracted from E14 rat embryos of female Sprague‐Dawley rats according to methods described previously.^[^ ^9^, ^14^ ^]^ Briefly, following decapitation under anesthesia induced by inhalation of isoflurane, embryonic sacs were extracted and placed in ice cold Hanks blank salt solution. The embryos were then carefully removed from their sacs, ventral mesencephalons were dissected from the brains and mechanically dissociated with a pipette until a homogenous cell suspension was obtained. Cells were then seeded on poly‐l‐lysine‐coated plain glass slides (2947‐75 × 38, Corning, Dublin, Ireland), where three division score‐marks were previously engraved, each subdivision received 300 000 cells. The slides were then placed in a 100 mm Petri‐dish (CLS430293, Corning, NY, USA) covered by 10 mL of DMEM/F12 (D6421, Sigma‐Aldrich, Dublin, Ireland) supplemented with 6 g L^−1^ of d‐glucose, 100 µg mL^−1^ of Primocin (InvivoGen, Toulouse, France), 2 mm of l‐glutamine (Sigma, Wicklow, Ireland), 10 mL L^−1^ of FBS and 20 mL L^−1^ of B27 supplement (17504‐044, Gibco, NY, USA), which was replaced every two days until their utilization in different experiments.

### Oscillatory Fluid Shear Stimulation

Oscillatory fluid shear stress (OFS) was applied using a PPFC previously designed and optimized as described by Stavenschi et al.^[^ ^154^, ^155^, ^156^ ^]^ Briefly, a glass slide (75 × 38 × 1 mm) covered with cells was placed between two plates and an oscillatory pressure‐driven fluid flow was propelled through it using 5 mL syringes (Terumo) mounted on a programmable double syringe pump (AL‐4000; World Precision Instruments, Hertfordshire, UK). Flow rates of 22.45 and 4.49 mL min^−1^ at 0.5 Hz were used to obtain a shear stress magnitude of 0.5 and 0.1 Pa, respectively. The VM cells were lysed for protein, GAGs and RNA extraction or fixed for immunocytochemical staining after 1, 7, and 14 days postexposition to fluid shear stress for 1, 4 or 6 h (Figure S3, Supporting Information). Under static control conditions cell cultures were similarly maintained within the chambers but were not subjected to OFS.

### Immunocytochemistry

Cells were fixed for 15 min using a 4% paraformaldehyde solution, followed by three washes in 1× phosphate buffered saline (PBS) and a 5 min permeabilization process using a chilled buffer containing 0.103 g mL^−1^ sucrose, 2.92 mg mL^−1^ NaCl, 0.6 mg mL^−1^, MgCl_2_, 4.76 mg mL^−1^ HEPES buffer, and 0.1% Triton X‐100 in water at pH 7.2. Nonspecific targets were blocked at 37 °C for 30 min using PBS containing 1% bovine serum albumin (BSA). Primary antibodies (see Table S1, Supporting Information, for suppliers and dilutions) were incubated overnight at 4 °C on a rotary shaker at 100 rpm. The next day, cells were washed three times with PBS‐T 0.05%. Fixed samples were incubated with secondary antibodies (Table S1, Supporting Information) for 1 h and protected from the light at RT under slow shaking. Samples were then washed two times for 5 min with PBS 1× and coverslip mounted in Fluoroshield with DAPI (Sigma, F6057).

### In Vitro Image Analysis

Z‐stack images were obtained at a 60× magnification using an FV1000 Fluoview Confocal Laser Scanning Biological Microscope (Olympus, Dublin, Ireland). All acquisition settings were held constant, allowing intensity quantification and comparison. A minimum of five fields of view (FOV) was acquired per technical replicate, resulting in ≈30 images per control and experimental groups. Images were processed using ImageJ (W. RasBand, National Institute of Health, Bethesda, USA). The stacks obtained from each FOV were projected into a single image using the maximal intensity projection, then the individual channels were isolated and quantified separately. Intensity, area and number of positive cells were automatically counted after thresholding the image as previously described by Healy et al.^[^ ^157^ ^]^ For each specific stain, the best automatic thresholding methods were assessed using the “Threshold Check” plugin. Specifically, the following methods were used to convert the image to binary: renvy and yen for astrocytes, moments, and maxentropy for microglia, li, and mean for neurons, minimum for DAPI. Obtained values were normalized either by the staining area or according to the total cell number in the FOV, automatically quantified with the DAPI signal.

### Electrode Implantation In Vivo Study

Research and animal procedures were approved by the UCD Animal Research Ethics Committee (AREC 17‐22) and licenses by the Health Product Regulatory Authority of Ireland (AE18982‐P122).

Deep brain stimulation electrodes were implanted into the left subthalamic nucleus (STN) (Figure S8A, Supporting Information) of seven male Wistar rats 9 weeks old and weighing 385 g (±15.4 g) at the time of surgery. Prior to surgery, rats were housed in stable pairs for a minimum of 1 week, in a controlled environment pathogen free facility, with a 12/12 h light‐dark cycle regime, access to water and standard rodent diet ad libitum, a temperature of 21.7 °C (±0.017 °C) and humidity of 48.8% (±0.195%). Cages were cleaned three times a week and contained paper shreds, woodchip bedding, and wooden balls or sticks as enrichment.

On the day of surgery, rats were brought to the procedure room and anaesthetized using 4.5% isoflurane in 4 L min^−1^ oxygen, the depth of anesthesia was verified with pedal withdrawal and corneal reflex prior to 1.2–1.8% isoflurane in 1 L min^−1^ oxygen for anesthesia maintenance. Antibiotics (Metronidazole (20 mg kg^−1^, QD) and Gentamicin (6 mg kg^−1^, QD)), and analgesia (Buprenorphine 0.015–0.03 mg kg^−1^, BID) were administrated subcutaneously preoperatively. For body temperature stability, rats were laid on a heating blanket at 37 °C in the aseptically prepared surgical field throughout the entire surgery. Next, rats were placed on a Stoelting stereotaxic frame (Dublin, Ireland) using nonrupture ear bars and local anesthetic cream (Emla 5% cream, AstraZeneca, Cambridge, UK) on both rat ears and frame bars. Eyes’ dryness was avoided by using a tear replacement ointment (Vidisic, Dr. Gerhard Mann, Chem.‐pharm. Fabrik GmbH, Berlin, Germany). Prior to incision, a maximum of 0.5 mL of Lidocaine at 0.5% (diluted from Lidocaine 1%, Hameln Pharmaceuticals Ltd, Gloucester, UK) was injected subcutaneously prior skin incision. From between the eyes to between the ears an incision was drawn to expose the skull and visualize Bregma and Lambda. Four Stoelting screws (1.59 mm) were anchored into the skulls, 2 in the frontal bone on the left and right of the bregma and 2 in the parietal bone on the left and right of the lambda. Left and right STN coordinates (location −3.6 DV and −2.5 mm ML from Bregma and −7.6 mm from the dura) were determined after corrections for the differences in rat skull size, when needed, and the 2 insertion holes were drilled using a Stoelting burr until appearance of the dura. The dura was carefully sectioned, and SNEX‐100 electrode (Microprobes for Life Science, Gaithersburg, USA) mounted on the stereotaxic frame and slowly descended to the STN location. Once in place, the electrode was secured to the skull using cyanoacrylic glue (Loctite, Henkel, Germany) and dental cement (Dentalon plus, Heraeus Kulzer GmbH, Hanau, Germany) was used to cover the entire skull and the anchoring screws, promoting a state of relative electrode micromotion at the electrode/tissue interface. In four rats an electrode was slowly implanted into the midbrain and then withdrawn from the subthalamic nucleus to create a stab incision. Finally, the skin was sutured both cranially and caudally around the cement cap using intradermal suture (Vycyl 4‐0, Ethicon Inc., Somerville, USA).

The surgeries lasted ≈2 h and the rats were returned to their home cage for recovery with a heating blanket beneath the cage and soft food for the first 18 h. After recovery, rats were single‐housed for up to 24 h to recover and then reintroduced to their initial partner. The antibiotic course was given until 4 days postoperation and the dose of analgesic was adjusted according to the observed pain level as measured using the Rat Grimace Score.^[^ ^158^ ^]^

### Immunohistochemistry of Brain Tissue

At 8 weeks postelectrode implantation, animals were anesthetized with 5% isoflurane in 4 L min^−1^ oxygen and once rats were unresponsive to tail/toe pinches, animals were perfused transcardially with PBS followed by 10% formalin (Sigma‐Aldrich, Arklow, Ireland), after administration of heparine (625 IU per rat, INNOHEP 2500 IU, LEO Pharma, Ballerup, Denmark). Rats’ brains were then removed and postfixed in a 4% PFA solution at 4 °C for at least 24 h prior removal of the probe from the brain. Fixed brains were soaked in a 15% sucrose solution overnight at 4 °C, followed by a second equilibration in a 30% sucrose solution overnight at 4 °C until the brain dropped to the bottom of the vial. Using a brain matrice, tissues were then finely dissected to obtain a cube containing the subthalamic nucleus and paraffin‐embedded using the Thermo Scientific Excelsior ES Tissue Processor. Tissue blocks from a total of 7 animals (*n* = 6 electrode group; *n* = 4 stab wound group; *n* = 3 craniotomy/healthy tissue group) were perpendicularly sectioned to the electrode tract into 10 µm thick sections using a Leica RM2135 microtome. Consecutive sections were mounted on SuperFrost Plus slides allowing tracking of the electrode *z*‐axis and direct comparison between sections.

After deparaffination in a xylene bath for 2 × 5 min, tissue sections were rehydrated through increased ethanol concentration baths (100%; 100%; 90%; 70%) for 2 min each, followed by a final water bath for 5 min. Sections were then subjected to an antigen retrieval protocol, incubated in a Tris/EDTA solution (10 mm/1 mm; pH9) in a pressure cooker during 20 min. Next, tissue sections were permeabilized using a chilled buffer (0.103 g mL^−1^ sucrose, 2.92 mg mL^−1^ NaCl, 0.6 mg mL^−1^, MgCl_2_, 4.76 mg mL^−1^ HEPES buffer, and 0.1% Triton X‐100 in water; pH 7.2) for 5 min and nonspecific targets were blocked for 30 min with a diluent solution containing 3% of BSA and 0.1% Tween‐20 in PBS (PBS‐T). Following blocking, sections were incubated with primary antibodies to visualize either neuron nuclei (NeuN), astrocyte cytoskeleton (GFAP) or microglia cell body (Iba1) in diluent buffer at 4 °C overnight (see Table S1, Supporting Information, for suppliers and dilutions). After three washes of 5 min in PBS‐T, the corresponding secondary antibodies (Table S1, Supporting Information) were incubated in diluent buffer for 1 h at RT. Finally, sections were washed three times for 5 min with PBS, before being counterstained and coverslip mounted with Fluoroshield with DAPI (Sigma, F6057). The researcher performing the tissue staining remained blinded to the treatment groups.

### Quantitative Brain Tissue Analysis

Tissue section images were obtained at 20× magnification using an FV1000 Fluoview Confocal Laser Scanning Biological Microscope (Olympus, Dublin, Ireland). All acquisition settings remained identical across all sections allowing intensity quantification and comparison. Images were processed using ImageJ (W. RasBand, National Institute of Health, Bethesda, USA). Three to six sections were imaged and analyzed per animal, resulting in 18 to 36 sections per control and experimental groups. For in vivo quantification with respect to the distance from the electrode insertion site, ROI boxes with a constant width of 200 µm and length of 50 µm were used every 50 µm and up to 250 µm radially outward from the probe void. The mean intensity and percentage of the stained area was measured in each ROI box for GFAP and Iba1 stained sections and NeuN^+^ nuclei were counted using the “analyze particles” ImageJ tool. For each NeuN stained image, the distance of the 6 nearest NeuN^+^ nuclei from the electrode void was measured using the draw line tool. Finally, data were averaged for each group and plotted by mean ± SEM as a function of distance from the electrode site. The researcher performing the quantitative tissue analysis was blind to the different group identities.

### Western Blot Analysis

Total protein from VM cell populations were harvested using 100 µL of RIPA buffer (R0278, Sigma‐Aldrich, Dublin, Ireland) supplemented with 1% of protease inhibitor (Roche, Basel, Switzerland), phosphatase inhibitor cocktail I and III (Sigma‐Aldrich, Dublin, Ireland) and detached using a cell scraper (Sarstedt, Wexford, Ireland). The lysate was then centrifuged for 15 min at 14 000 rpm and 4 °C, the supernatant collected and stored at −70 °C. Sample protein concentrations were measured using Pierce BCA Protein Assay Kit (23227, Thermo Scientific, Waltham, USA) and 4× Laemmli sample buffer containing 200 mm of DTT (11583786001, Sigma‐Aldrich, Dublin, Ireland) was added to 10 µg of protein before being denatured at 95 °C for 5 min. All samples were then separated through a 10% SDS‐PAGE gel at 120 V and then transferred to 0.45 µm pore size nitrocellulose blotting membranes (10600002, Amersham Protran) using a Trans‐Blot Turbo Transfer System (Bio‐Rad, Watford, UK). Membranes were blocked in 5% skimmed milk dissolved in Tris buffered saline (TBS) for 1 h, followed by incubation with 1:1000 primary antibodies dissolved in 5% milk‐TBS‐Tween 20 (T‐TBS) overnight at 4 °C. Blots were incubated with HRP‐conjugated secondary antibodies at 1:10 000 for 1 h at RT (see Table S1, Supporting Information, for suppliers) and developed with SuperSignal West Pico PLUS Chemiluminescent Substrate (ThermoFisher, Waltham, USA) on CL‐X Posure X‐ray films (34091, ThermoScientific, Waltham, USA). Protein relative expression was quantified through band densitometry.

### Protein Antibody Microarray

Nexterion slide H microarray slides were acquired from Schott AG (Mainz, Germany). CF 555, succinimidyl ester was purchased from Sigma‐Aldrich (SCJ4600022, Dublin, Ireland).

The protein–antibody array was constructed as previously described.^[^ ^9^, ^159^, ^160^, ^161^, ^162^ ^]^ A total of 42 commercial antibodies (see Table S2, Supporting Information, for catalogue numbers and dilution detail) were buffer‐exchanged with PBS and quantified using the Pierce BCA Protein Assay Kit (23227, Thermo Scientific, Waltham, USA). Approximately 1 nL was printed per feature on Nexterion H amine‐reactive hydrogel‐coated glass slides using a SciFLEXARRAYER S3 piezoelectric printer (Scienion, Berlin, Germany). The antibodies were maintained at 20 °C in a 62% relative humidity environment during printing. Each microarray slide contained eight replicate subarrays, with each antibody spotted in replicates of six per subarray. After printing, slides were incubated in a humidity chamber overnight at RT to facilitate complete antibody‐slide conjugation. Residual functional groups were deactivated by immersion in 100 mm ethanolamine in 50 mm sodium borate, pH 8.0, for 1 h at RT. Slides were washed 3× in PBS/0.05% Tween 20 (PBS‐T) for 2 min, followed by 1 wash in PBS. Slides were dried by centrifugation (470 × *g*, 5 min) prior to storage with desiccant at 4 °C until use.

Protein fluorescent tagging was carried out using CF 555, succinimidyl ester according to manufacturer's instructions. Briefly, the 1 mg vial of CF 555 was resuspended in 100 µL of DMSO and 4 µL of CF 555 solution was added in 50 µL of protein sample and 50 µL of boric acid. All the samples were left to incubate for 1 h at RT. The excess fluorescent probe was removed through molecular weight and the buffer was exchanged with PBS (pH 7.4) using 3 kDa centrifugal filter units (UFC500396, Amicon Ultra – 0.5 mL, 3 kDa, Sigma‐Aldrich, Dublin, Ireland). Absorbance was measured at 555 and 280 nm for all tagged samples and calculations were performed according to manufacturer's instructions using an arbitrary extinction coefficient of 100 000 and molecular mass of 100 000, allowing quantification of relative protein concentration and label substitution efficiency.

Prior to use, the antibody microarray slides were allowed to equilibrate to RT for 30 min under desiccant. Fluorescently labeled protein lysates were incubated on microarrays as previously described^[^ ^159^, ^160^, ^161^, ^163^ ^]^ with limited light exposure throughout the process. Initially, two labeled samples were titrated (2.5 to 10 µg mL^−1^) to determine the optimal concentration for all samples to obtain an extractable, nonsaturated signal response (i.e., N1000 and 65 000 relative fluorescence units (RFU)) with low background (b500 RFU) for all samples (Figure S5, Supporting Information). In brief, 70 µL of each sample diluted to a concentration of 7.5 µg mL^−1^ in TBS‐T was applied to each well of the microarray and incubated for 1 h at 23 °C in the dark on a horizontal shaker (4 rpm). After incubation, slides were washed 3× for 2 min in TBS‐T, 1× in TBS and then centrifuged until dry. Once dried, microarray slides were scanned immediately using a G2505 microarray scanner (Agilent Technologies, Santa Clara, CA, USA) using a 532 nm laser (5 µm resolution, 90% laser power).

Microarray data extraction was performed as previously described.^[^ ^159^, ^160^, ^162^, ^164^ ^]^ In short, GenePix Pro v6.1.0.4 (Molecular Devices, Berkshire, UK) was used to extract raw intensity values from image files using a proprietary *.gal file which enabled the identification of 230 µm printed protein spots using adaptive diameter (70–130%) circular alignment. The data were then exported to Excel (version 2010, Microsoft). Local background‐corrected median feature intensity data (F633 median‐B633) values were selected, and the median of six replicate spots per subarray was handled as a single data point for graphical and statistical analysis. Hierarchical clustering of normalized data was performed using Hierarchical Clustering Explorer v3.0 (http://www.cs.umd.edu/hcil/hce/hce3.html) using the parameters: no prefiltering, complete linkage, and Euclidean distance.

### DNA Content

DNA content was measured using a Quant‐iT PicoGreen dsDNA Assay Kit (Invitrogen, Dublin, Ireland) following manufacturer's instruction. Briefly, 28.7 µL of DNA suspension or Lambda DNA standard (provided by the manufacturer) was added to wells with 100 µL of 1× TE buffer (10 mm Tris‐HCl, 1 mm EDTA, pH 7.5) and 71.3 µL of 1× Quant‐iT PicoGreen working solution. The fluorescent intensity was then read with a spectrophotometer using an excitation wavelength of 480 nm and an emission wavelength of 520 nm.

### Quantitative Real‐Time PCR

Total RNA from shear flow treated and static control cultures was extracted using the phase separation method. Briefly, the VM culture of two technical replicate slides were detached in 250 µL of TRI reagent (Sigma) using a cell scraper (Sarstedt, Wexford, Ireland) and were pooled together. Samples were transferred in a nuclease‐free tube and stored at −80 °C until RNA extraction. Later, thawed samples were vigorously shaken and vortexed followed by a 5 min RT incubation to ensure full cell lysis and entire protein and nucleic acid complex dissociation. Next, 100 µL of 1‐Bromo‐3‐chloropropane was added to each sample, vigorously shaken for 15 s and left 5 min to incubate at RT before being centrifuged at 17 000 × *g* for 15 min at 4 °C. The upper transparent RNA phase was carefully retrieved without disturbing the DNA phase to avoid any genomic contamination, this entire step was repeated three times to ensure a maximal RNA yield for each sample. Then, an equivalent volume of 2‐propanol (≈200 to 300 µL) was added to the collected phase in addition of 1 µL of GlycoBlue (Invitrogen) (a blue nucleic acid coprecipitant allowing a better visualization of the RNA pellet), samples were agitated and incubated for 5 min at RT or 30 min at −20 °C, before centrifugation at 17 000 × *g* for 30 min at 4 °C to obtain total precipitation of the RNA pellet. The supernatant was carefully discarded and nuclease‐free 70% EtOH was used to wash the pellet with moderate shaking followed by centrifugation at 7500 g for 5 min at 4 °C. A second wash step was repeated with 100% EtOH. The RNA pellet was left to dry at RT for 5 min and resuspended in 14 µL of THE RNA storage solution (Invitrogen) supplemented with 1 µL Protector RNase Inhibitor (Roche). The RNA concentration was measured using a NanoDrop 2000c Spectrophotometer (ThermoFischer) and the purity and quality was verified using a 260/280 and 260/230 nm absorbance ratio, only samples with a ratio between 1.8 and 2.2 were kept for cDNA synthesis.

To avoid false amplification, the genomic DNA from each sample was eliminated prior cDNA synthesis, both steps were performed using the RT^2^ First Strand Kit (Qiagen) and following manufacturer's instructions, all steps were carried out on ice. First, 2 µL of the GE buffer was mixed with 450 ng of RNA, the volume was adjusted to 10 µL with RNase‐free water and incubated at 42 °C for 5 min, constituting the DNA elimination mix. Next, the RT^2^ First Strand Kit reverse transcription mix was prepared with 4 µL of 5× BC3 buffer, 1 µL of control P2, 2 µL of RE3 reverse transcriptase mix, and 3 µL of RNase‐free water. Next, 10 µL of the reverse transcription mix was added to each DNA elimination mix, tubes were gently homogenized, and incubated for cDNA synthesis at 42 °C for 15 min in a Veriti Gradiant Thermal Cycler (Applied Biosystem). The reaction ended with a temperature rise at 95 °C for 5 min to inactivate the reverse transcriptase enzyme. Finally, the newly synthesized cDNA was diluted in 91 µL of RNase‐free water and stored at −80 °C.

Gene expression from ventral mesencephalic cell populations exposed to oscillatory and static control fluid flow conditions was pooled from four biological replicates and analyzed via qRT‐PCR using a Qiagen RT^2^ Profiler PCR Array Rat Neurogenesis and RT^2^ Profiler PCR Array Rat Hippo signaling pathway (GeneGlobe ID: PARN‐404Z and PARN‐172Z, respectively). Following manufacturer's instructions, the 384 wells of the array were filled with a 10 µL mix of nucleotide‐free water, RT^2^ SYBR Green qPCR Mastermix (Qiagen) and samples cDNA and the quantitative RT‐PCR was carried out with a LightCycler 480 (Roche). Results were quantified by a comparative Ct method and normalized using the five housekeeping genes included in the array.

### Extracellular Flux Analysis

The OCR and ECAR of VM cells cultured with the PIEZO1 antagonist GsMTx4 and the PIEZO1 agonist Yoda1 was measured using the Seahorse Cell Mito Stress Test with the extracellular flux Seahorse XFp analyzer (Agilent), in order to assess the mitochondrial respiration and glycolysis, respectively.^[^ ^165^, ^166^ ^]^ Briefly, VM cells were extracted and seeded in PLL‐coated Seahorse XF cell culture plates (25 000 cells per well), after 5 days of culture growth and differentiation, cells were exposed to either 10 µm of DMSO, 10 µm of Yoda1 or 0.5 µm of GsMTx4 in culture medium or the culture medium only (control) for 14 days with the medium and drugs refreshed every 2 days. On the 14th day of treatment, as per manufacturer's instructions, 1 h prior to flux analysis, samples were placed at 37 °C in a non‐CO_2_ incubator and the media replaced by Seahorse Base XF medium supplemented with 1 mm sodium pyruvate, 2 mm l‐glutamine and 10 mm glucose at pH 7.4 for CO_2_ and O_2_ equilibration. During this preincubation, the four drugs necessary to perform the Cell Mito Stress Test were loaded into the XF Sensor Cartridges with a preoptimized concentration of 2 µm for the oligomycin, 1 µm of rotenone and antimycin A (provided already mixed by the manufacturer to be loaded and injected together) and 3 µm of carbonyl cyanide‐4 (trifluoromethoxy) phenylhydrazone. All the OCR and ECAR values were normalized by DNA content measured for each well using the Quant‐iT PicoGreen dsDNA Assay Kit (Invitrogen, Dublin, Ireland) as previously described.^[^ ^167^ ^]^

### Statistical Analysis

All data of this work represent at least three biological replicates for each of the test groups and the control group. Data are presented as the mean of the values ± standard error of the mean. Statistical significance was determined by One‐way or Two‐way ANOVA followed by Tukey post hoc test to determine the statistical significance (*p* < 0.05), unless otherwise stated. All statistical analyses were performed using Minitab Express version 1.5.2.

## Conflict of Interest

The authors declare no conflict of interest.

## Author Contributions

T.W. and E.B. contributed equally to this work. A.W.: conceptualization, writing the original draft, data curation, and writing, reviewing, editing, formal analysis, investigation, and methodology. E.B.: formal analysis, data curation, investigation, and methodology. T.W.: formal analysis, data curation, investigation, and methodology. J.E.: formal analysis, data curation, investigation, and methodology. E.P.: formal analysis, data curation, investigation, and methodology. M.L.: resources, writing, reviewing, and editing. M.K.: resources, writing, reviewing, and editing. U.F.: supervision, resources, writing, reviewing, and editing. M.B.: supervision, funding acquisition, resources, project administration, conceptualized, writing the original draft, reviewing, and editing.

## Supporting information

Supporting Information

## Data Availability

The data that support the findings of this study are available from the corresponding author upon reasonable request.
