# Supplementary material for: Micromotion Derived Fluid Shear Stress Mediates Peri‐Electrode Gliosis through Mechanosensitive Ion Channels
Source: Adv Sci (Weinh). 2023 Jul 30;10(27):2301352. doi: 10.1002/advs.202301352 (PMC10520674; doi:10.1002/advs.202301352)
Supplement: Supplementary file 1 — Supporting Information [file ADVS-10-2301352-s001.pdf]

ADVANCED SCIENCE
Open Access

Supporting Information

for *Adv. Sci.*, DOI 10.1002/advs.202301352

Micromotion Derived Fluid Shear Stress Mediates Peri-Electrode Gliosis through Mechanosensitive Ion Channels

*Alexandre Trotier, Enrico Bagnoli, Tomasz Walski, Judith Evers, Eugenia Pugliese, Madeleine Lowery, Michelle Kilcoyne, Una Fitzgerald and Manus Biggs**

Supporting Information

Micromotion derived fluid shear stress mediates peri-electrode gliosis through mechanosensitive ion channels

*Alexandre Trotier, Enrico Bagnoli+, Tomasz Walski+, Judith Evers, Eugenia Pugliese, Madeleine Lowery, Michelle Kilcoyne, Una Fitzgerald, Manus Biggs**

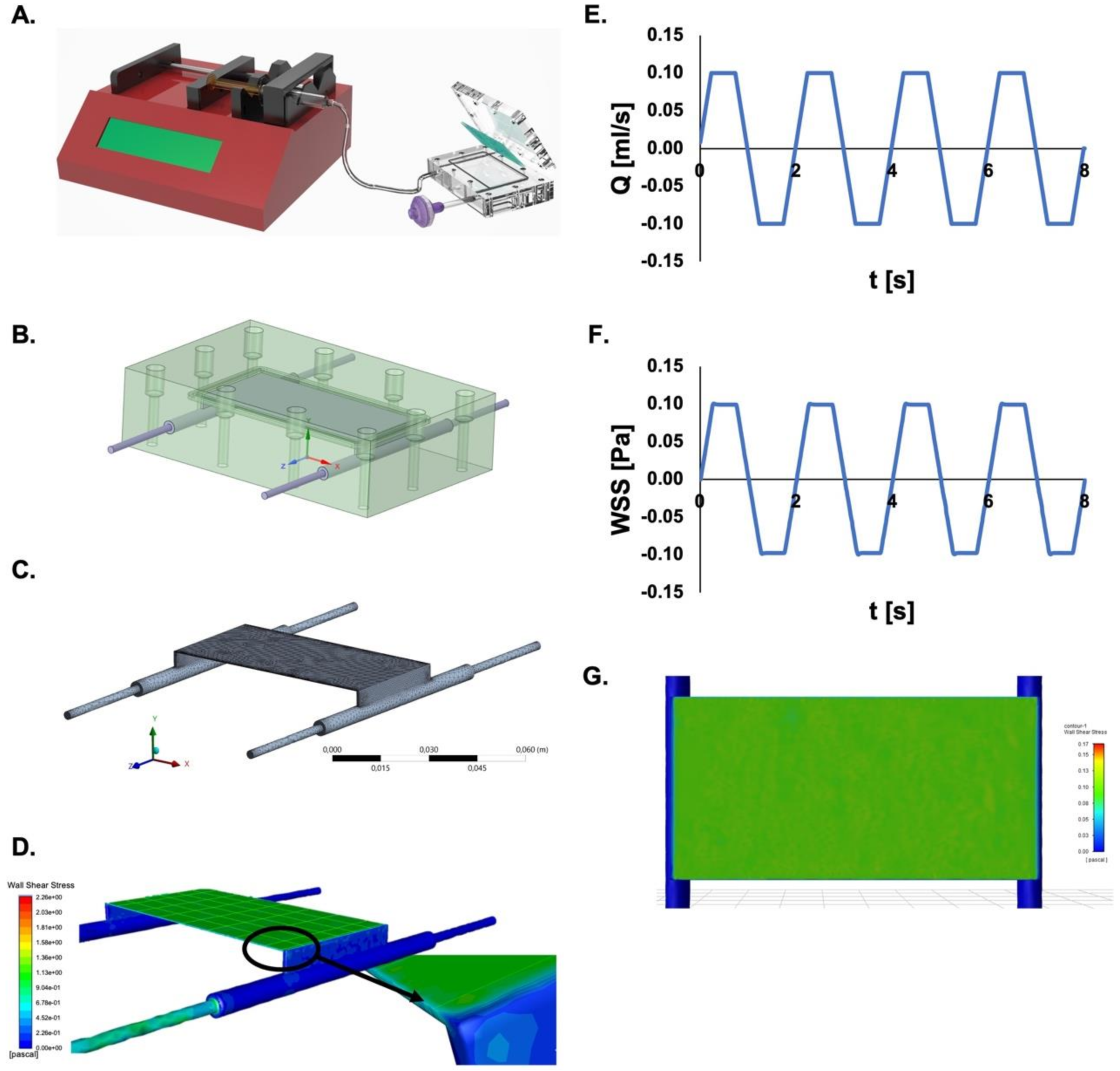

Figure S1. Parallel plate flow chambers induce homogeneous and consistent oscillatory fluid flow shear stresses on neural cell monolayers. General concept of the setup providing oscillatory fluid stimulation conditions to VM cultures including PPFCs enclosing a glass slide covered with a cell monolayer connected to a syringe-pump which controls the flow direction and rate **(A)**. The 3D design of the PPFC geometry, where green represents solid components and violet denotes fluid domain **(B)**. The mesh of the fluid volume used for numerical calculations **(C)**. WSS distribution on the surface of the PPFC with sampling lines for the assessment of the coefficient of variance **(D)**. Changes in oscillatory volumetric flow rate at the inlet of the PPFC **(E)** with accompanying changes of WSS measured at the central point of the microchannel surface **(F)** for Q = 4.49 ml/min. The homogeneity of WSS on the top surface of the microchannel for Q = 4.49 ml/min during the steady flow period **(G)**.

Table S1. Summary of test conditions and results of computational studies of the WSS homogeneity in the developed PPFC system.

Q [ml/min]	4,49	44,89	380,46
WSS [Pa] mean	0.098	0.990	9.70
WSS [Pa] SD	0.008	0.079	0.83
CV	8.4%	8.0%	8.6%
p-value*	< 0.0001	< 0.0001	< 0.0001

* Results of the two-tailed unpaired t-test comparison of the WSS values on the microchannel surface (full) vs. the surface reduced by the edges area corresponding to the decreased values of the WSS (reduced).

Table S2. Antibody source and dilution used for immunohistochemistry

Antibody	Host/Isotype	Supplier	Catalogue No.	Dilution
**Primary**				
GFAP	Mouse mAb	Sigma	G3893	1:500
β-Tubulin III	Rabbit pAb	Sigma	T2200	1:500
Chondroitin Sulfate	Mouse mAb	Sigma	C8035	1:200
AQP4	Rabbit pAb	Alomone	AQP-004	1:500
PIEZO1	Rabbit pAb	Alomone	APC-087	1:200
TRPA1	Rabbit pAb	Alomone	ACC-037	1:200
NeuN	Rabbit mAb	Abcam	ab177487	1:1000
Iba1	Rabbit pAb	Wako	019-19741	1:500
**Secondary (Alexafluor)**				
Anti-mouse 488	Donkey pAb	Invitrogen	A-21202	1:500
Anti-Rabbit 594	Donkey pAb	Invitrogen	A-21207	1:500

Table S3. Summary of all commercial antibodies used for the construction of the antibody microarray

Order	Probe	Concentration (mg/mL)	Species	Company	Ref
1	Integrin beta5	0,35	Rabbit	Cell signalling	D24A5
2	P-Smad1	0,25	Rabbit	Cell signalling	S206 D40B7
3	P-p44/42 MAPK	0,05	Rabbit	Cell signalling	T202/Y204 (197G2)
4	b-catenin 200 ug	1	Rabbit	Millipore	06-734
5	Anti-Active-b-Catenin (anti ABC) Antibody, clone 8E7	1	Mouse	Sigma®	05-665-25UG
6	Integrin beta1 [EPR1040Y] 100ul ((0.157 mg/ml)	1	Rabbit	Abcam	ab134179
7	Collagen I [EPR7785] 100 ul (0.875 mg/ml)	1,003	Rabbit	Abcam	ab138492
8	Anti-Mouse IgG H&L	0,05	Goat	Abcam	ab150113
9	FAK 100ug	0,547	Mouse	MBL	12G4
10	BMPR1A (PA5-11856)	1	Rabbit	ThermoFisher	PA5-11856
11	Collagen II [2B1.5] 250 ul (0.2 mg/ml)	0,25	Mouse	Abcam	ab185430
12	Myelin [MBP101] 100 ug (3.2 mg/ml)	0,125	Mouse	Abcam	ab62631
13	Collagen V 100 ug (1mg/ml)	0,856	Rabbit	Abcam	ab7046
14	SCXA (100ug)	0,1	Rabbit	Abcam	ab58655
15	Biglycan (100ug)	0,183	Rabbit	Abcam	ab49701
16	TBHS4	1,086	Rabbit	Abcam	ab176116
17	Tenascin C (50ug)	0,1	Rabbit	Abcam	ab88280
18	Decorin	1,075	Rabbit	Abcam	ab175404
19	Tenomodulin (100ug)	0,5	Rabbit	Abcam	ab203676
20	Collagen III 100 ug (1mg/ml)	0,002	Rabbit	Abcam	ab7778
21	Olig2 [EPR2673]	0,25	Rabbit	Abcam	ab109186
22	KCNK4 100 ul (0.5mg/ml)	0,002	Rabbit	Abcam	ab81367
23	Integrin beta 3 [crc54]	0,1	Rabbit	Abcam	ab34409
24	P-FAK	0,002	Rabbit	Cell signalling	Y925
25	p-smad1/5	0,1	Rabbit	Cell signalling	S463/465
26	p44/42 MAPK (ERK 1/2)	0,1	Mouse	Cell signalling	L34F12
27	Smad1	0,05	Rabbit	Cell signalling	D59D7
28	Smad5	0,004	Rabbit	Cell signalling	D4G2
29	Anti-beta Actin antibody	0,75	Mouse	Abcam	ab8226
30	Anti-Glial Fibrillary Acidic Protein (GFAP)	0,25	Mouse	Sigma®	G3893
31	Nestin (Rat-401)	0,5	Mouse	SantaCruz®	sc-33677
32	CD81 (H-121)	1	Rabbit	SantaCruz®	sc-9158
33	Integrin alphaM (OX42)	1	Mouse	SantaCruz®	sc-53086
34	Phosphate buffered saline, pH 7.4				

35	Paxillin antibody [Y113]	0,5	Rabbit	Abcam	ab32084
36	cleaved spectrin alpha II (h1186)	0,5	Rabbit	SantaCruz®	sc-23464
37	Anti-Smad3 antibody [EP568Y]	1	Rabbit	Abcam	ab40854
38	Anti-Chondroitin Sulfate antibody	0,1	Mouse	Sigma®	C8035
39	Phosphate buffered saline, pH 7.4				
40	VR1 (H-150)	1	Rabbit	SantaCruz®	sc-20813
41	Anticorps L-type Ca++ CP α1C (H-280)	0,5	Rabbit	SantaCruz®	sc-25686
42	PIEZO1 Antibody (N-15)	1	Goat	SantaCruz®	sc-164319
43	PIEZO2 Antibody (G-20)	1	Rabbit	SantaCruz®	sc-84763
44	ANKTM1 (C-19)	0,5	Goat	SantaCruz®	sc-32353
45	TREK-1 Antibody (C-20)	1	Goat	SantaCruz®	sc-11557
46	Anti-β-Tubulin III	0,75	Rabbit	Sigma®	T2200

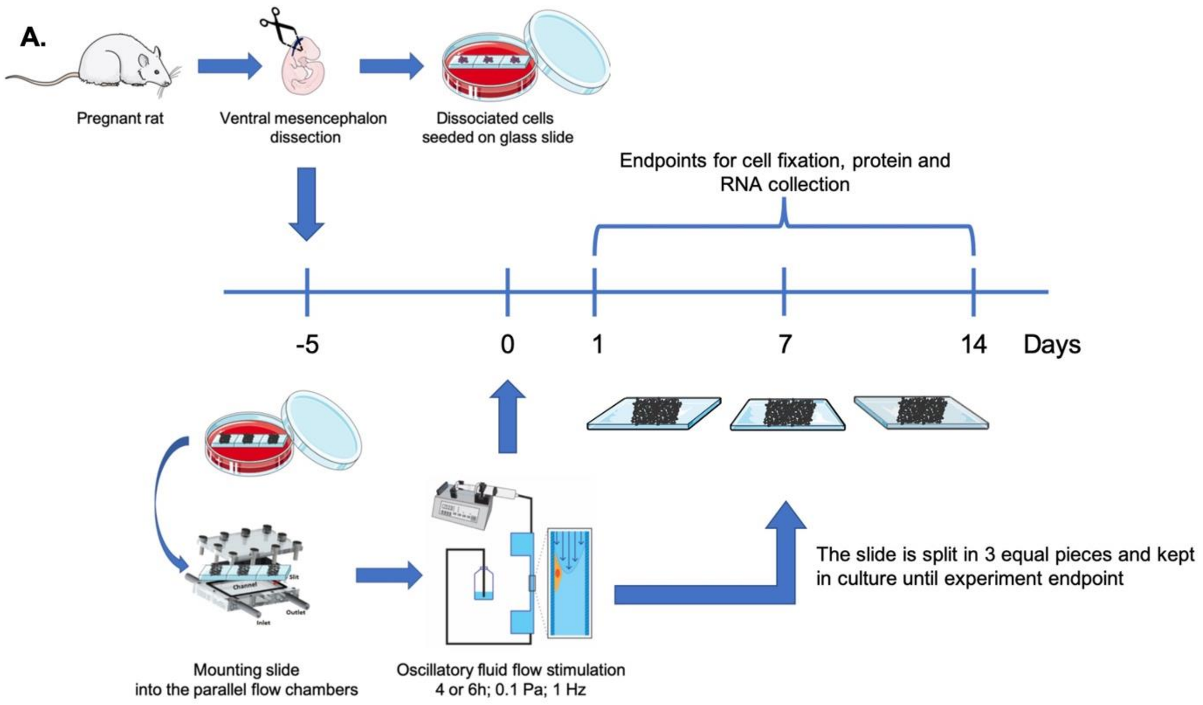

Figure S2. Study timeline and setup. Primary neural cells were extracted from the ventral mesencephalon of E14 rat embryos and seeded onto a glass slide five days prior to exposure to shear stress. At day 0, the glass slide was placed in the parallel flow apparatus and exposed to an oscillatory fluid flow at 0.1 Pa, 0.5 Hz for either 4 or 6h using pulsed culture medium. Following shear stimulation, glass slides were subdivided into 3 equal pieces, and kept in culture until experiment endpoint for either fixation or RNA/protein extraction.

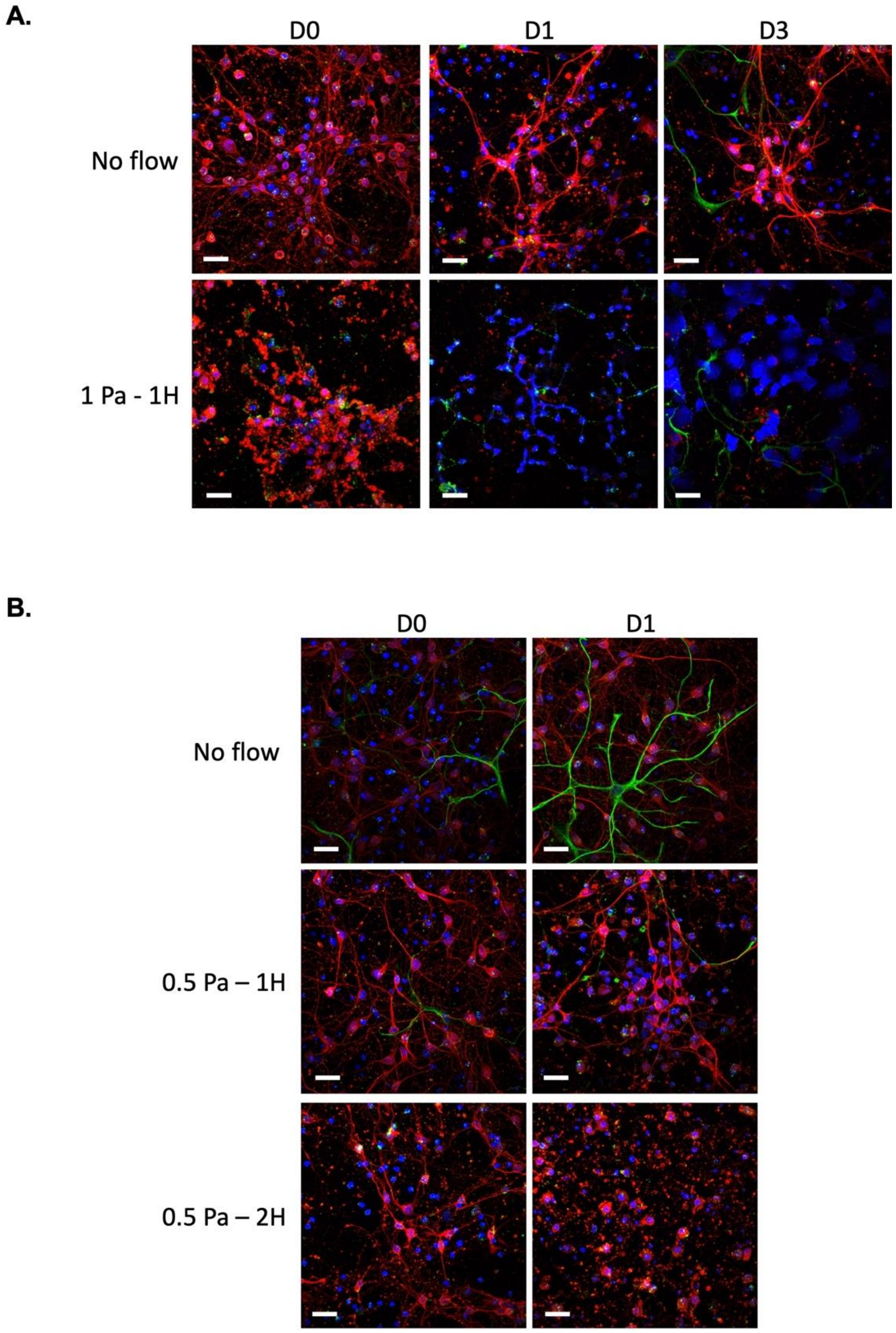

Figure S3. The effect of oscillatory fluid flow shear stress magnitude (Pa) and duration on neuron viability. Ventral mesencephalic cells did not survive shear stresses of 1 Pa for 1h **(A)** or 0.5 Pa for 2h **(B)**. VM cultures were immunofluorescently stained for GFAP (green), nucleus (blue) and ß-Tubulin III (red) immediately and/or 1 and/or 3 days post-stimulation (scale bar = 20 µm; n=3). Indeed, at day 0 immediately following exposure to 1 Pa fluid-flow for 1h, the cytoplasm of all the cells appeared to have burst and the culture did not recover over the 3 next days in culture **(A)**. Similarly, after exposure to fluid flow conditions of 0.5 Pa for 2h, VM culture viability deteriorated at day 0 in comparison to cells cultured under static control conditions and this deterioration was exacerbated leading to complete culture loss by day 1 post-stimulation **(B)**. Scale bar = 20 µm.

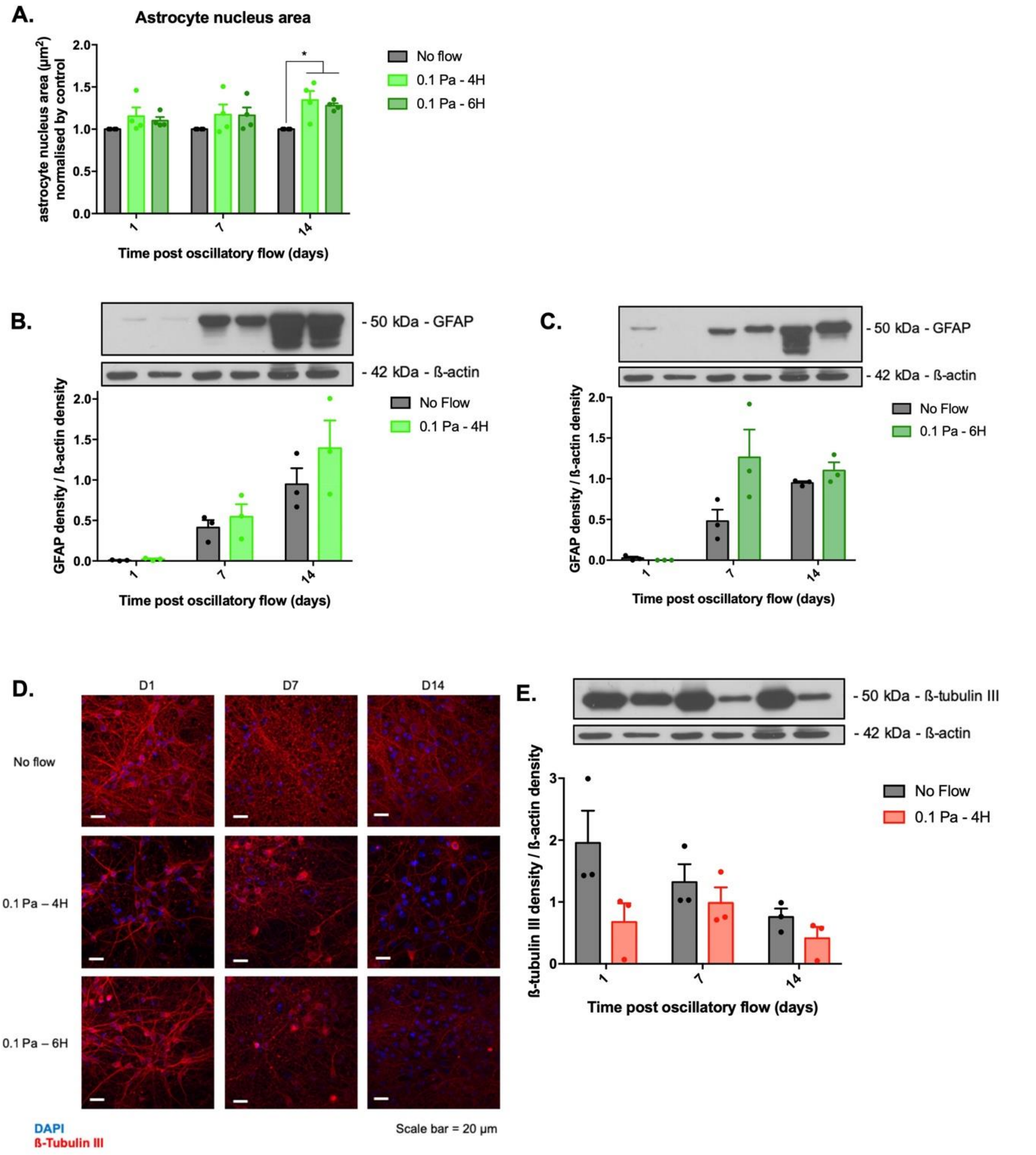

Figure S4. Oscillatory fluid flow shear stress promotes a reactive astrocyte phenotype and neuron death in ventral mesencephalic (VM) cells. The mean astrocyte nuclear area also increased by 15 to 25% following exposure to shear stress at all time points **(A)**. GFAP upregulation was confirmed by western blotting where flow shear stress induced an overexpression of total GFAP after 7 and 14 days post stimulation **(B,C)**. VM cells stained for nucleus (blue) and ß-Tubulin III (red) **(D)** (scale bar = 20 µm; n=3). The effects of shear stress on the neuron viability were confirmed by western blotting, ß-Tubulin III total protein exhibiting reduction at all time points following VM exposure to 4h shear stress at 0.1 Pa **(D)**. Data are represented as mean ± SEM (n=3-4). One-way ANOVA with Tukey post hoc test was performed. *, **, *** represents a statistically significant difference ($p<0.05$), ($p<0.01$) and ($p<0.001$), respectively.

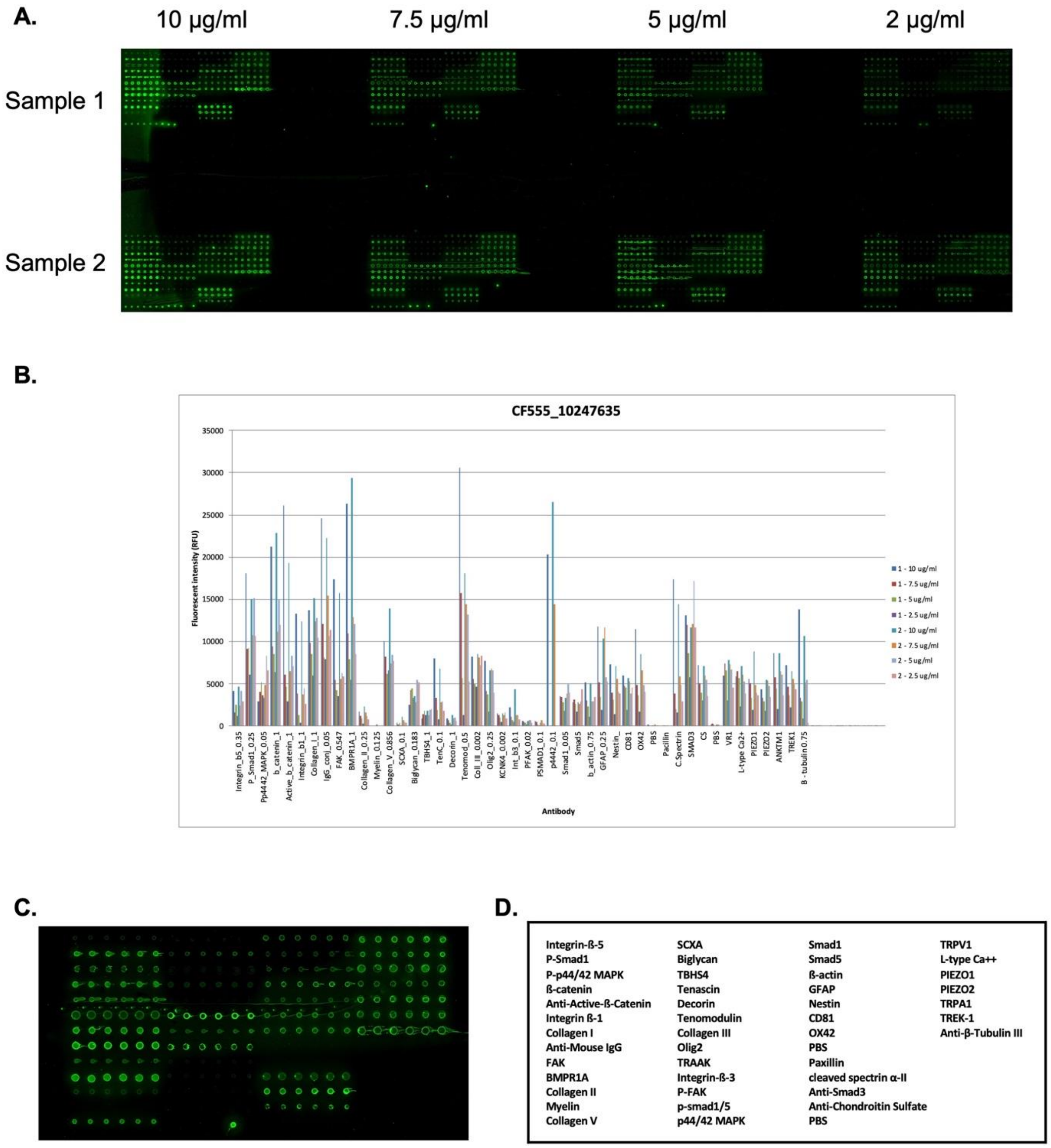

Figure S5. Optimisation of the antibody microarray. Two representative protein samples obtained from VM cell lysates were labelled using CF™ 555 and incubated on a microarray slide at four different concentrations (10; 7.5; 5 and 2.5 µg/ml before optical scanning **(A).** Image analysis was performed to plot the fluorescence intensity of printed antibodies **(B),** in order to select the optimal protein concentration leading to the highest fluorescence reading without reaching saturation. A concentration of 7.5 µg/ml **(C)** was found optimal and sample 1 was systematically incubated as an internal control for all subsequent microarray analysis to ensure consistent printing and robust antibody performance. Each subarray antibody was printed with 6 technical replicates as detailed in **(D)**.

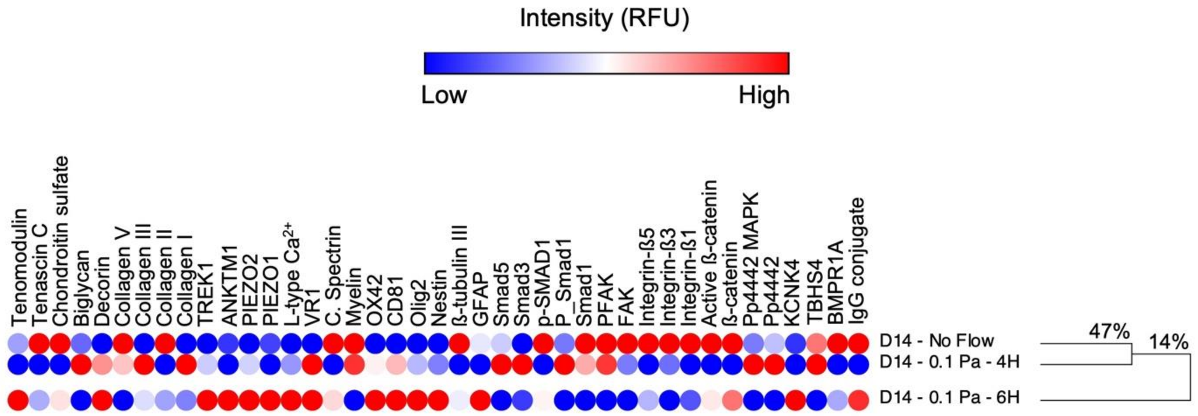

Figure S6. Hierarchically clustered heatmap depicting binding intensities of whole cell lysate to the custom antibody protein microarray (n=4). Data clustering analysis identified a similarity level of 47% in VM cells exposed to static control conditions and VM cells exposed to 4h of shear flow, while indicating a 14% similarity level in the expression of these proteins in VM cells exposed to 6h of shear flow.

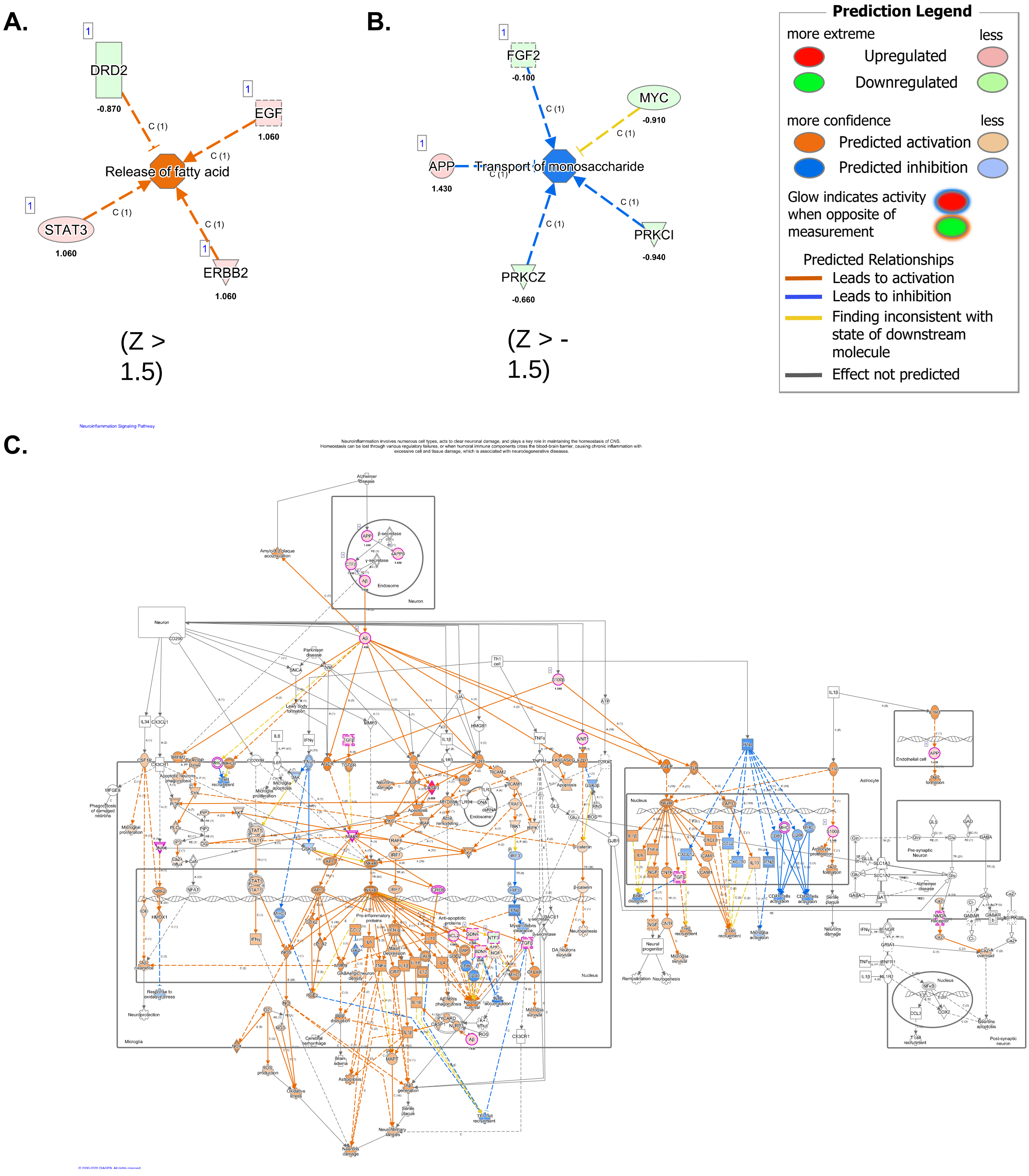

Figure S7. Ingenuity Pathway Analysis predicted the activation of the neuroinflammation canonical pathway and biological functions related to neural metabolism. The Qiagen® IPA software predicted the activation of two additional biological function networks related to metabolism: the function “release of fatty acid” was predicted as undergoing activation, with an activation Z>1.5 **(A)**, whereas the function “transport of monosaccharide” was detected as being inhibited with a Z< -1.5 **(B)**. Moreover, the Qiagen® IPA software also predicted the overall activation of the neuroinflammation pathway, with a Z-score> 2, including 24 genes which underwent statistically significant modulation **(C)**.

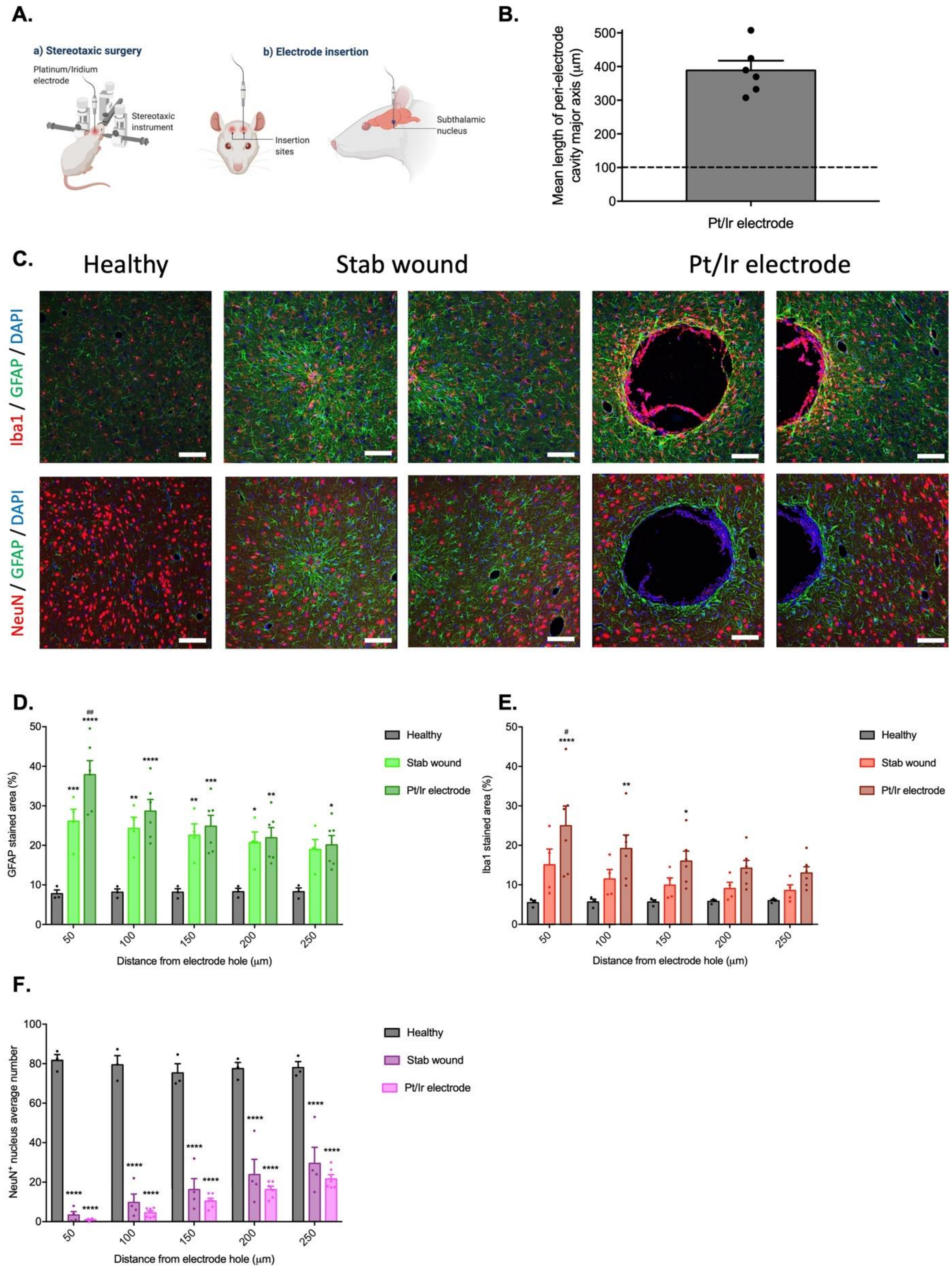

Figure S8. Subthalamic electrode implantation and stab wound injury lead to the development of a glial scar *in vivo*. Schematic detailing the previously established rat model of stab injury or Pt/Ir electrode implantation into the subthalamic nucleus for 8 weeks **(A)** (created with BioRender.com). Quantification of the cavity cross-section shows an average ellipse major axis length ~4 times greater than the electrode diameter (100 µm; represented by a dashed line) **(B)**. To confirm that electrode implantation initiated glial scarring,

immunofluorescent staining of GFAP (green), Iba1 (red), NeuN (red) and DAPI (blue) were performed on the processed brain tissue sections (scale bar = 100 µm) **(C)**. Quantification of the GFAP stained area in both the stab injury and implanted condition revealed a significant increase in astrocyte reactivity relative to the healthy control condition as a function of implantation site distance **(D)**. Similarly, quantification of the Iba1 staining area indicated significant increases in as far as 150 µm from the electrode implantation region **(E)**. Conversely, the number of mature neuron nuclei (NeuN^{+}) was significantly reduced at distances up to 250 µm in both the stab injury or implanted experimental groups relative to the healthy control group **(F)**. Data are represented as mean ± SEM (n=3-6). Two-way ANOVA with Tukey post hoc test was performed. *, **, *** represents a statistically significant difference versus the healthy control and #, ##, ### versus the stab wound condition, (p<0.05), (p<0.01) and (p<0.001), respectively.

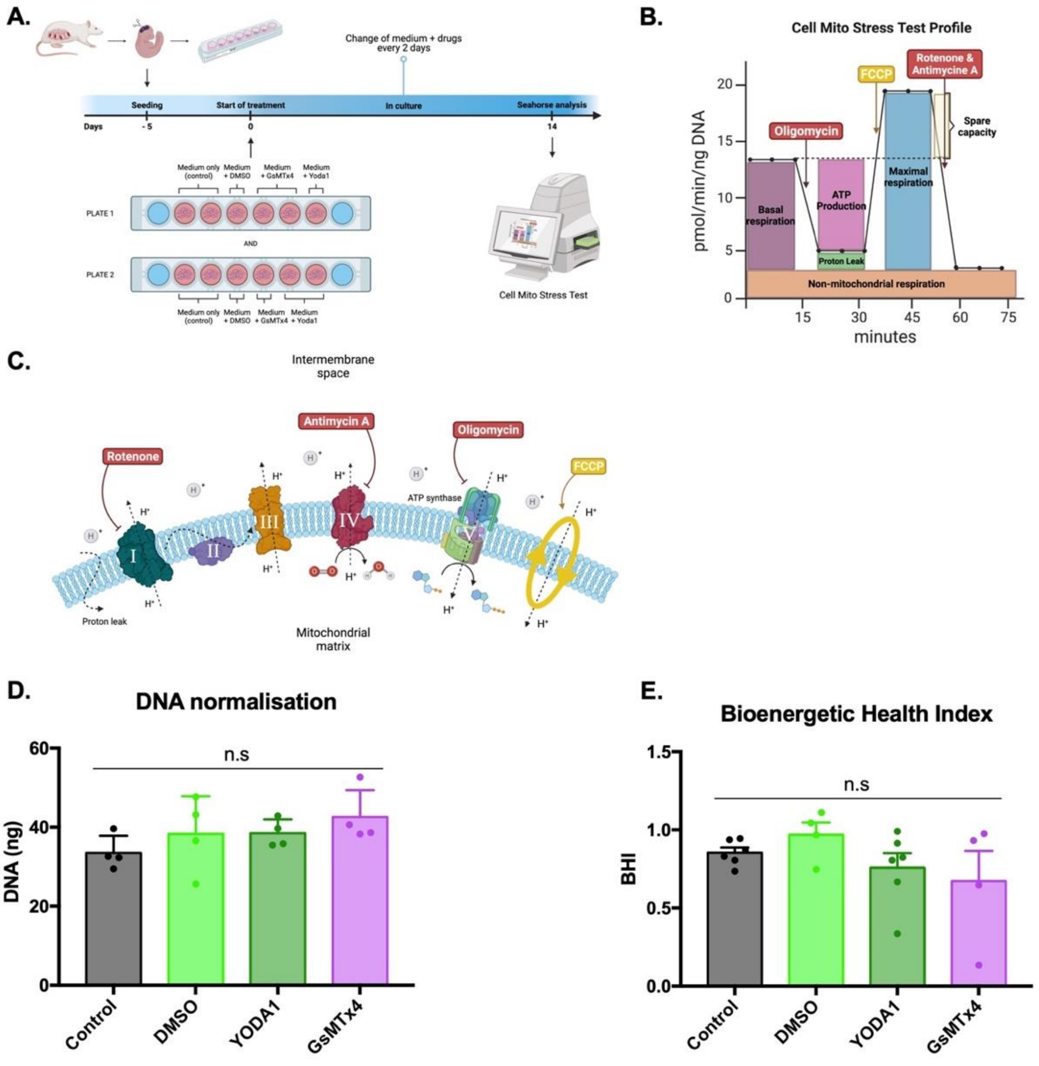

Figure S9. Effect of the PIEZO1 receptor antagonist (GsMTx4) and agonist (Yoda1) on mitochondria health. Schematic of the experiment design and timeline **(A).** VM cells were extracted and seeded into Seahorse XF cell culture plates, , after 5 days of culture cells were exposed to either medium only (control), medium and DMSO, medium and GsMTx4 or medium and Yoda1 and kept in culture for 14 days with media and drug replenishment every 2 days. The Cell Mito Stress Test was performed after 14 days using the Seahorse XF flux

analyser. Graph representing the typical oxygen consumption rate (OCR) profile of healthy cells during the Mito Stress test and mitochondrial functions (Basal respiration, ATP production, Maximal respiration, Spare capacity, Non-mitochondrial respiration and Proton leak) which can be directly calculated from these readings are displayed as well as the injection time of the different assay molecules **(B)**. Schematic of the mechanisms of the 4 molecules used in the Mito Stress Test **(C)**. Initially, cells are exposed to oligomycin, an inhibitor of the ATP synthase (complex V) which creates a drop in ATP production by reducing the electron flow through electron chain transport (ETC), leading to a decreased mitochondrial respiration (OCR), this difference in respiration level corresponds to the ATP production. Conversely, subsequent exposure to Carbonyl cyanide-4 (trifluoromethoxy) phenylhydrazone (FCCP), an uncoupling agent, re-establishes the ETC by collapsing the proton gradient and disrupting the mitochondrial membrane potential, promoting a significant increase in mitochondrial OCR, corresponding to the maximal respiration. The difference between the basal respiration and maximal respiration allows the calculation of the spare capacity value which corresponds to the mitochondria capacity to respond to increased metabolic demand. Final dual exposure to Rotenone and Antimycine A, inhibitors of complex I and III respectively, significantly inhibits mitochondrial respiration facilitating calculation of the non-mitochondrial respiration value, as well as proton leak. All graph and schematics were created with BioRender.com. All flux analysis were normalized to the DNA content (ng) of each well **(D)**. The bioenergetic health index calculation, indicator of the mitochondria health, showed no significant differences between both control and experimental groups **(E)**. Schematics created with BioRender.com.

Table S4. Modulation summary table of cellular markers of glial scarring and mechanosensitive ion channel in response to oscillatory fluid flow following 14 days *in vitro* or 8 weeks post-electrode implantation *in vivo*.

		Astrocytes	Neurons	Microglia	Other glial scar markers		Mechanosensitive ion channels	
In vitro		**GFAP**	**ß-tubulin III**	**Iba1**	**CS**	**AQP4**	**PIEZO1**	**TRPA1**
**0.1 Pa – 4H**		↑	=	NA	↑	=	=	=
**0.1 Pa – 6H**		↑	↓↓	NA	↑	↑	↑	↑
**No flow + GsMTx4**		↑↑↑	↓↓↓	NA	NA	NA	NA	NA
**No flow + YODA1**		=	=	NA	NA	NA	NA	NA
**0.1 Pa – 6H + GsMTx4**		↑↑↑↑	=	NA	NA	NA	NA	NA
**0.1 Pa – 6H + YODA1**		↓↓	=	NA	NA	NA	NA	NA
In vivo		**GFAP**	**NeuN**	**Iba1**	**CS**	**AQP4**	**PIEZO1**	**TRPA1**
**Stab**	**50 µm**	↑↑↑	=	=	NA	NA	↑↑↑	=
	**250 µm**	=	↓↓↓↓	=	NA	NA	↑↑↑	=
**Implant**	**50 µm**	↑↑↑↑	↓	↑↑↑↑	NA	NA	↑↑↑↑	↑
	**250 µm**	↑	↓↓↓↓	=	NA	NA	↑↑↑↑	=

↑ and ↓ indicates statistically significant increase or decrease of either stained cell number, protein expression or staining area, = indicates no statistical difference of neither stained cell number, protein expression or staining area, with respect to static or non-implanted control conditions.
